# The fast-recycling receptor Megalin defines the apical recycling pathway of epithelial cells

**DOI:** 10.1038/ncomms11550

**Published:** 2016-05-16

**Authors:** Andres E. Perez Bay, Ryan Schreiner, Ignacio Benedicto, Maria Paz Marzolo, Jason Banfelder, Alan M. Weinstein, Enrique J. Rodriguez-Boulan

**Affiliations:** 1 Margaret Dyson Vision Research Institute, Department of Ophthalmology, Weill Medical College of Cornell University, 1300 York Avenue, New York, New York 10065, USA; 2 Departamento de Biología Celular y Molecular, Facultad de Ciencias Biológicas, Pontificia Universidad Católica de Chile, Avenida del Libertador Bernardo O'Higgins 340, 8331010 Santiago, Chile; 3 Department of Physiology and Biophysics, Weill Medical College of Cornell University, 1300 York Avenue, New York, New York 10065, USA; 4 Department of Cell and Developmental Biology, Weill Medical College of Cornell University, 1300 York Avenue, New York, New York 10065, USA

## Abstract

The basolateral recycling and transcytotic pathways of epithelial cells were previously defined using markers such as transferrin (TfR) and polymeric IgA (pIgR) receptors. In contrast, our knowledge of the apical recycling pathway remains fragmentary. Here we utilize quantitative live-imaging and mathematical modelling to outline the recycling pathway of Megalin (LRP-2), an apical receptor with key developmental and renal functions, in MDCK cells. We show that, like TfR, Megalin is a long-lived and fast-recycling receptor. Megalin enters polarized MDCK cells through segregated apical sorting endosomes and subsequently intersects the TfR and pIgR pathways at a perinuclear Rab11-negative compartment termed common recycling endosomes (CRE). Whereas TfR recycles to the basolateral membrane from CRE, Megalin, like pIgR, traffics to subapical Rab11-positive apical recycling endosomes (ARE) and reaches the apical membrane in a microtubule- and Rab11-dependent manner. Hence, Megalin defines the apical recycling pathway of epithelia, with CRE as its apical sorting station.

## Abstract (web summary)

Basolateral recycling and transcytotic pathways in epithelial cells are defined by specific markers, however the apical recycling pathway is poorly understood. Perez Bay *et al*. show that Megalin is a marker for this pathway, which intersects with the other routes in shared perinuclear recycling endosomes.

## Graphical abstract

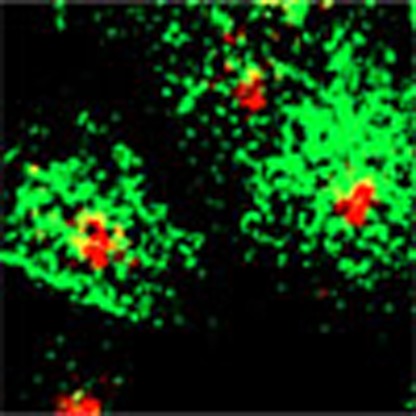

Megalin (gp330, LRP-2) is a member of the low-density lipoprotein receptor family, expressed exclusively in embryonic and adult general and neuro-epithelial cells, in which it mediates the endocytosis of a vast array of ligands. Knock-out of Megalin in mice causes a range of neuro-developmental abnormalities that result in perinatal death^1^, ostensibly because Megalin participates in the endocytosis and transcytosis of key differentiation factors, for example, sonic hedgehog^2^. Megalin also plays key roles in adult physiology. In the kidney, a 1:1 complex of Megalin and Cubilin (Fig. 1a) on the apical plasma membrane (PM) of proximal tubule (PT) cells binds and mediates endocytosis of a myriad of ultrafiltrate proteins (that is, hormone, vitamin and iron carriers, enzymes and immunoglobulin light chains)^3^,^4^,^5^, for subsequent lysosomal degradation and retrieval of their ligands and constituent amino acids into the blood^6^. Given that kidney filtration of the blood results in 180 l per day (refs 7, 8) of glomerular ultrafiltrate containing 10–30 g l^−1^ of low-molecular weight proteins^6^,^9^, Megalin and Cubilin are required to internalize a large amount of ultrafiltrate proteins to prevent their loss in urine^10^,^11^. Megalin-deficient mice display proteinuria and develop bone defects due to deficient internalization of vitamin D binding protein by PT cells^12^. In human genetic syndromes such as Donnai–Barrow/Facio–Oculo–Acustico–Renal Syndrome^13^, Stickler-like syndrome^14^ and Imerslund–Gräsbeck disease^15^,^16^, mutations in Megalin or Cubilin impair protein absorption in the kidney PT and the affected patients display proteinuria.

The endosomal itinerary of Megalin in polarized epithelial cells remains fragmentary as, surprisingly, most studies on Megalin endocytosis and recycling have been carried out in non-epithelial cell models. Experiments carried out in L2 (rat yolk sac carcinoma) cells have shown that Megalin reaches the same early endosomal compartments visited by the canonical fast-recycling marker transferrin receptor (TfR) in CHO and HeLa cells, that is, sorting endosomes (SE) and perinuclear Rab11-positive recycling endosomes (RE^Rab11^) (ref. 17, 18, 19) (Fig. 1b). However, epithelial cells exhibit a more complex endosomal architecture than non-epithelial cells, with separate apical sorting endosomes (ASE) and basolateral sorting endosomes (BSE), perinuclear Rab11-negative common recycling endosomes (CRE) and a subapical Rab11-positive endosomal compartment termed apical recycling endosome (ARE)^20^,^21^ (Fig. 1c). Studies in MDCK cells have clearly established that the fast-recycling basolateral receptors TfR and LDLR enter BSE and reach CRE, where they are sorted towards the basolateral PM through clathrin-coordinated interactions between their sorting signals and the clathrin adaptor AP-1B^22^,^23^,^24^,^25^. Likewise, the polymeric IgA receptor (pIgR) reaches CRE after internalization from the basolateral PM into BSE, but instead of recycling basolaterally, it is sorted apically by *N*-glycan apical signals through the ARE and to the apical PM^26^,^27^; a similar pathway is followed by TfR in AP-1B-deficient MDCK cells (Fig. 1c)^26^,^27^,^28^,^29^,^30^,^31^,^32^. Hence, CRE is a major sorting station at the intersection of well-defined basolateral recycling and transcytotic pathways. In contrast, only fragmentary information is available on a potential apical recycling pathway in epithelial cells and, in particular, on the recycling route of Megalin a candidate user of this pathway. For example, it has been reported that in LLC-PK1 kidney epithelial cells Megalin reaches ASE and exits this compartment in a PI3K-dependent manner^33^, whereas in MDCK cells, it co-localizes with Rab11 at steady-state^34^. However, it is not known if Megalin, after leaving ASE utilizes CRE as a major recycling station to the apical PM or, alternatively, is diverted directly to ARE for apical delivery. Furthermore, whereas TfR is known to recycle every ∼15 min to the PM for a total of ∼100 rounds^35^, it is still unclear whether Megalin is a long-lived receptor that undergoes many recycling rounds to the PM or is destroyed in the lysosomal compartment as a ‘sacrificial receptor' after a single internalization round.

Here we report the kinetics of Megalin endocytosis, recycling and degradation, its complete endosomal itinerary and underlying molecular mechanisms in the prototype epithelial cell line MDCK. We carried out experiments in both fully polarized and subconfluent MDCK cells and dual-colour confocal live-imaging in the latter, as they display endosomal compartments equivalent to CRE and ARE (Fig. 1d)^28^,^30^, which are more easily resolvable by optical microscopy. We developed a mathematical model to describe the trafficking kinetics of Megalin between all endosomal stations of its recycling pathway and the PM. Our experiments show that Megalin is internalized into ASE, subsequently transported to CRE, where it is mixed with basolaterally internalized proteins and sorted towards ARE and recycled back to the apical plasma membrane in a microtubule- and Rab11-dependent manner. Our results define an apical recycling pathway that intersects the basolateral recycling and transcytotic pathways at CRE. They also demonstrate that Megalin is a long-lived, non-sacrificial receptor that rapidly recycles to the apical plasma membrane, which provides novel mechanistic insight on the high protein absorption capacity of the kidney PT and on genetic proteinuric syndromes.

## Results

### Megalin internalization recycling and degradation kinetics

**Figure 1:**
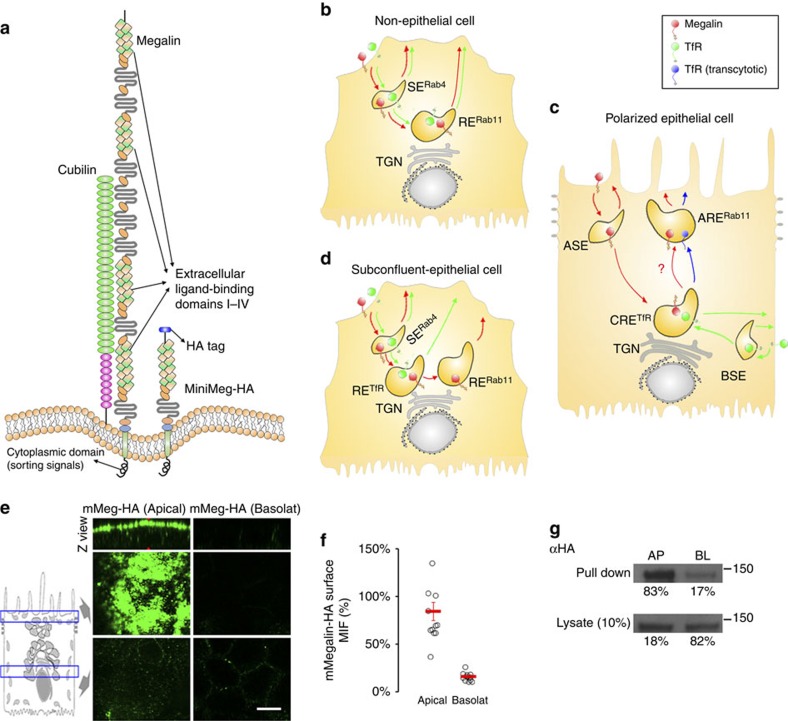
Model of Megalin and TfR recycling in epithelial and non-epithelial cells. (**a**) Molecular representation of endogenous Megalin,Cubilin and the mMeg-HA construct. mMeg-HA contains an HA tag in the luminal domain and the entire cytoplasmic tail bearing all trafficking signals (that is, two endocytic NPxY signals and one apical sorting signal NxxY). (**b**) Non-epithelial cells: both Megalin and TfR are internalized into peripheral SE, where a pool of these receptors is recycled to the PM and another is transported to perinuclear RE before recycling back to the PM. (**c**) Polarized epithelial cells: TfR is internalized from the basolateral PM into BSE, transported to CRE and either recycled to the basolateral PM in AP-1B-positive epithelia or transcytosed to ARE in AP-1B-negative epithelia. In contrast, Megalin is internalized from the apical PM into ASE, transported to CRE, mixed with basolaterally internalized TfR, sorted to ARE and recycled to the apical PM. (**d**) Subconfluent epithelial cells: most Megalin is sequentially transported through three endosomal compartments (SE^Rab4^>RE^TfR^>RE^Rab11^) before recycling back to the PM. In contrast, TfR is recycles through two endosomal compartments (SE^Rab4^>RE^TfR^). (**e**) mMeg-MDCK cells polarized on Transwell filters were incubated with 488-MαHA in the apical (left) or basolateral (right) chambers (45min at 4 °C), washed (15 min at 4 °C) and fixed. Panels show Z view (top) and two confocal sections in the apical (middle) and supranuclear regions (bottom). (**f**) MIF quantification of the domain selective immunofluorescence described in **e**, where circles represent individual cells and red lines represent average and standard error. (**g**) Western blot analysis of mMeg-HA expression from domain selective surface biotinylation followed by streptavidin immunoprecipitation in filter-polarized mMeg-MDCK cells. Pull down (surface) and Lysate (total) fractions are shown. Scale bar, 10 μm.

In order to study the trafficking kinetics and endosomal itinerary of Megalin in epithelial cells, we constructed MDCK cell lines stably expressing mini-Megalin (mMeg-MDCK cells), a truncated form of the protein lacking three of the four ligand-binding extracellular domains, tagged luminally with a HA epitope (mMeg-HA) (Fig. 1a). Because mMeg-HA contains the full cytoplasmic tail of Megalin, with all known Megalin sorting signals, it is believed to traffic like the full-length Megalin^36^,^37^,^38^; furthermore, it displays characteristic apical polarity in polarized MDCK cells, as revealed by domain selective immunofluorescence (Fig. 1e,f) and surface biotinylation (Fig. 1g).

**Figure 2:**
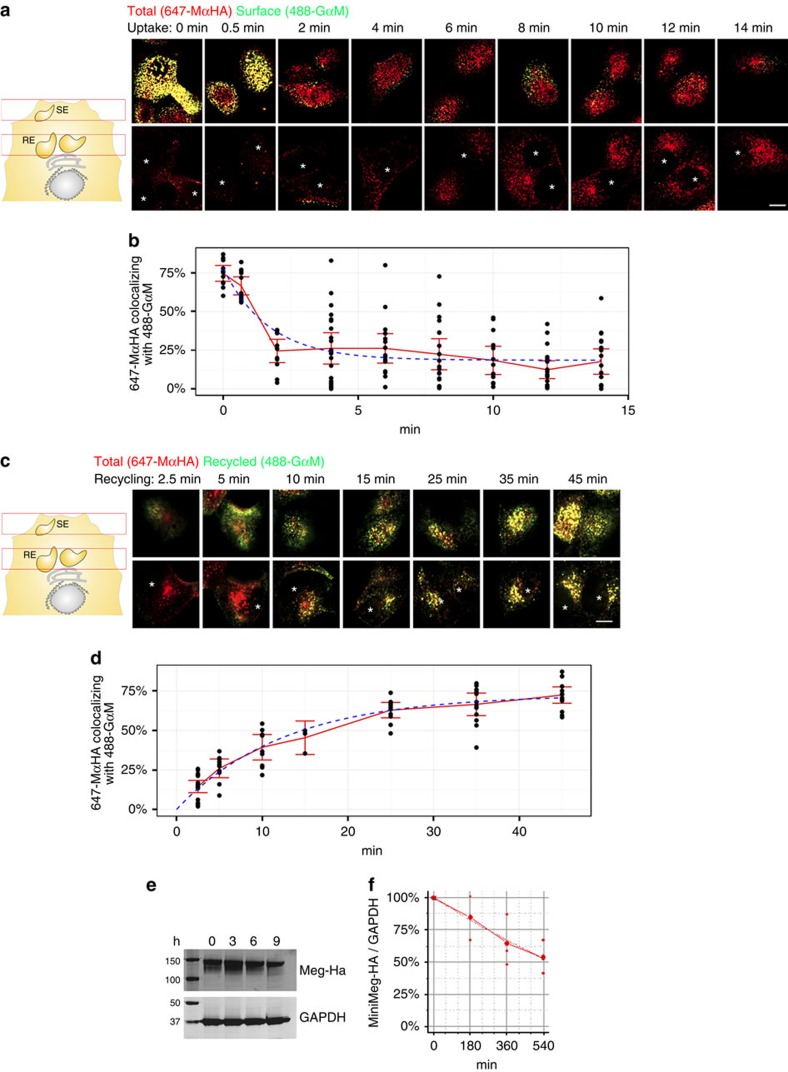
Megalin is endocytosed and recycled rapidly but degraded slowly in subconfluent MDCK cells. (**a**) Confocal images of subconfluent mMeg-MDCK cells allowed to internalize pre-bound 647-MαHA antibody at 37 °C for the indicated times, fixed and immunostained with 488-GαM without permeabilizing. Panel show confocal sections in the upper part of the cells (top) and the perinuclear region (bottom) of the respective cells. Asterisks indicate the nuclei. (**b**) Co-localization quantification and fitted curve of the percentage of the 647-MαHA pixels co-localizing with the 488-GαM pixels, which informs the percentage of the total labelled mMeg-HA localized at the PM at the indicated time points. Circles represent individual cells, the red line represent average and CI_95_ and the blue line represents the fitted curve. (**c**) Confocal images of subconfluent mMeg-MDCK cells allowed to internalize 647-MαHA antibody for 45 min, washed and subsequently allowed to recycle for the indicated times in the presence of 488-GαM. (**d**) Co-localization quantification and fitted curve of the percentage of the 647-MαHA pixels co-localizing with the 488-GαM pixels, which informs the percentage of total mMeg-HA recycled to the PM at the indicated time points. (**e**) Western blot analysis of mMeg-HA and GAPDH expression in subconfluent mMeg-MDCK cells treated with cycloheximide. (**f**) Quantification and fitted curve of the mMeg-HA/GAPDH ratio from two experiments as the one displayed in **c**. Scale bar, 10 μm.

We first measured the kinetics of Megalin endocytosis, recycling and degradation in MDCK cells (Fig. 2). To measure Megalin endocytosis (Fig. 2a,b), we labelled the PM mMeg-HA pool with a mouse anti-HA antibody tagged with SeTau-647 (647-MαHA)^29^, allowed internalization for the indicated times and labelled the remaining PM Megalin pool with secondary goat anti-mouse antibodies tagged with Alexa-488 (488-GαM) (for full protocol description, see Supplementary Fig. 1a). At *t*=0, virtually all 647-MαHA-decorated mMeg-HA localized at the PM as demonstrated by the almost complete colocalization with 488-GαM (Fig. 2a; see Supplementary Fig. 1b–d for split images). In striking contrast, after *t*=2 min, most 647-MαHA-decorated mMeg-HA was quickly internalized as it did not co-localize with 488-GαM. At *t*=8 min, 647-MαHA labelling started to concentrate in a perinuclear compartment, consistent with the reported trafficking of internalized Megalin to perinuclear recycling endosomes in non-epithelial cells^37^. Fitting of the colocalization data to a mono-exponential function (*y*=*y*_0_+*Ae*^−*x*/*t*^) (Fig. 2b)^39^ revealed that the internalization half time (*t*_1/2_) of Megalin was 1.2 min, with a 95% confidence interval (CI_95_) of 0.8−1.8 min. The fraction of 647-MαHA that co-localized with 488-GαM plateaued at 19% (CI_95_=14−22%), consistent with a 19% PM/ 81% intracellular steady-state distribution of Megalin, very similar to the 18% PM/80% total ratio observed in surface biotinylation assays in polarized MDCK cells (Fig. 1g).

To measure Megalin recycling to the PM (Fig. 2c,d), the fraction of previously internalized 647-MαHA returning to the cell PM was measured through the binding of 488-GαM, continuously added to the medium from *t*=0 (for full protocol description, see Supplementary Fig 2a). Parallel experiments determined that 647-MαHA does not dissociate from mMeg-HA after incubation with low pH medium, addressing concerns of its binding in the acidic endosomal environment (Supplementary Fig. 3). At *t*=2.5 min, most 647-MαHA localized intracellularly and only a small fraction (∼20%) co-localized with 488-GαM; however, at longer times, colocalization of 647-MαHA with 488-GαM increased, reflecting Megalin recycling to the PM (Fig. 2c; see Supplementary Figs 2b–d for split images). Fitting of the colocalization data to a mono-exponential function (*y*=*y*_0_+*Ae*^*x*/*t*^) revealed that Megalin recycling *t*_1/2_ was 9 min with a CI_95_ of 7–11 min (Fig. 2d).

To measure the kinetics of Megalin degradation, we used western blot with Ha antibodies in mMeg-MDCK cells exposed to cycloheximide for various times (Fig. 2e). Fitting the Megalin expression data to a mono-exponential function (Fig. 2f) showed that Megalin degradation *t*_1/2_ was 4.8 h (lower boundary of CI_95_=1.8 h), 32 × slower than its recycling time course (9 min), and similar to TfR degradation (Supplementary Figs 2f,g), a well-characterized fast-recycling, long-lived membrane receptor^29^,^40^.

In conclusion, Megalin is rapidly internalized and recycled, but slowly degraded, supporting the notion that, like TfR, Megalin is a fast-recycling receptor rather than a sacrificial receptor.

### Mathematical model of Megalin endosomal itinerary

As mentioned in the opening paragraph, previous studies have characterized the endosomal itinerary of basolateral receptors (TfR and LDLR)^22^,^23^,^24^,^25^ and the transcytotic receptor polymeric IgA (pIgR)^26^,^27^ in polarized and subconfluent MDCK cells. In contrast, the endosomal itinerary of Megalin in epithelial cells remains fragmented. As polarized and subconfluent epithelial cells display equivalent sets of endosomes (Fig. 1c,d), we decided to start by characterizing the endosomal itinerary of Megalin using live confocal imaging in subconfluent MDCK cells, where all endosomal compartments are in the same plane. This model allows to perform high-temporal resolution recording of the movement of Megalin between different compartments and to generate a basic mathematic model of the kinetics of these movements.

**Figure 3:**
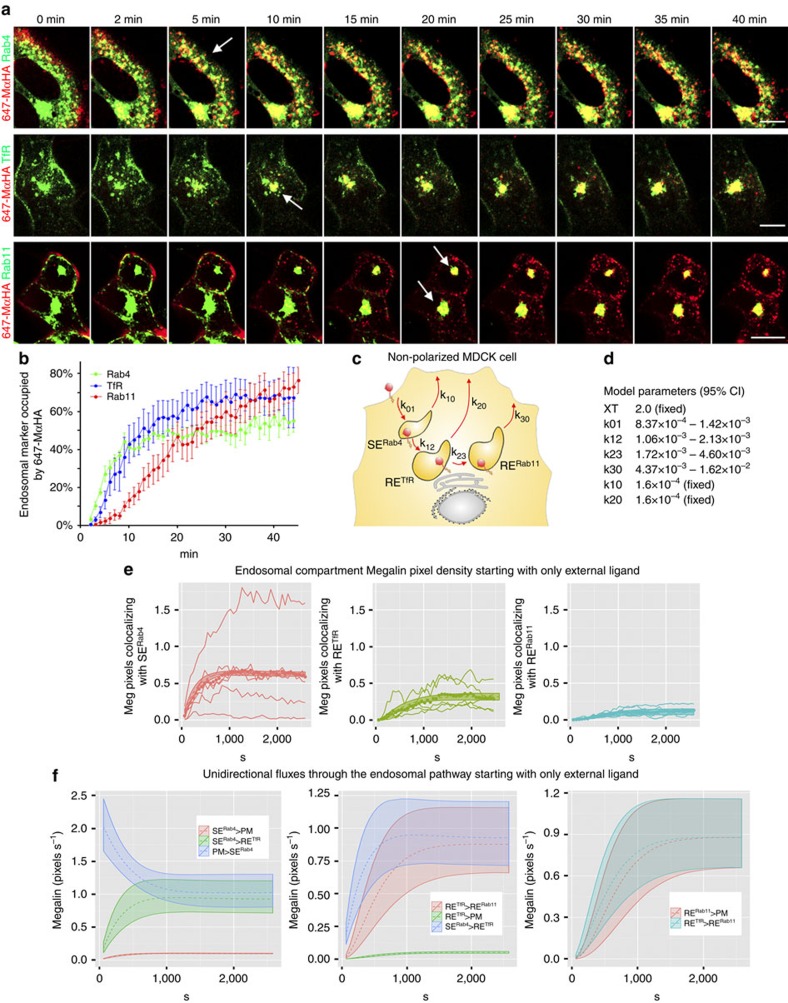
Mathematical model of Megalin endosomal itinerary in subconfluent MDCK cells. (**a**) Representative time-lapse confocal sections from 2D movies of subconfluent MDCK cells co-transfected with mMeg-HA and the SE marker Rab4-GFP (top), the RE markers TfR-GFP (middle) or Rab11-mCherry (bottom). Pre-bound 647-MαHA (45  min at 4 °C) was allowed to internalize (45 min at 37 °C) during live-imaging acquisition. (**b**) Co-localization time course for each endosomal marker with internalized 647-MαHA. Curves represent average and s.e. values from six movies per condition. (**c**) Cartoon displaying all endosomal compartments of subconfluent mMeg-MDCK cells and the constant coefficients (*k*_01_, *k*_10_, *k*_12,_ *k*_20_, *k*_23_ and *k*_30_) denoting the transfer rates for sequential movement of Megalin through these compartments. (**d**) Constant coefficients values (*k*) of the model. (**e**) Kinetics of Megalin pixels colocalizing with SE^Rab4^, RE^TfR^ and RE^Rab11^ for the experimental replicas (continuous curves), experimental averages (dots) and CI_95_ model prediction (shaded areas). (**f**) Unidirectional Megalin fluxes (*J*_*ij*_) across the PM, SE^Rab4^, RE^TfR^ and RE^Rab11^. Scale bar, 10 μm.

For these experiments, we co-transfected pairwise subconfluent MDCK cells with mMeg-HA and Rab4-GFP (SE^Rab4^), TfR-GFP (RE^TfR^) or Rab11-mCherry (RE^Rab11^). We decorated the PM pool of mMeg-HA with 647-MαHA at 4 ^o^C and recorded its endosomal trafficking after shift to 37 ^o^C by two colour spinning-disc confocal microscopy. Like TfR, mMeg-HA reached successively SE^Rab4^ and RE^TfR^; however, in striking contrast with TfR, mMeg-HA reached RE^Rab11^ subsequently to its appearance at RE^TfR^ (Fig. 3a, arrows and Supplementary Movies 1–3). The time course of the colocalization of internalized 647-MαHA with each endosomal marker (Fig. 3b) confirmed that the endosomal itinerary of Megalin in subconfluent MDCK cells is PM>SE^Rab4^>RE^TfR^>RE^Rab11^. Three-dimensional movies yielded identical results as two-dimensional movies (Supplementary Fig. 4 and Supplementary Movies 4–6).

To represent mathematically Megalin's endosomal itinerary we modelled the transfer rate coefficients (*k*) for the sequential movement of Megalin along SE^Rab4^, RE^TfR^ and RE^Rab11^ and between these compartments and the PM (Fig. 3c). To estimate the uncertainty in the optimized *k* values, a Markov Chain Monte Carlo (MCMC) based approach was used to generate an ensemble of credible sets of these parameters (Fig. 3d) (see Methods for model description). Figure 3e compares collected raw data with simulation results from an ensemble of credible models. The panels show the time course of Megalin colocalizing with SE^Rab4^ (left panel), RE^TfR^ (middle panel) and RE^Rab11^ (right panel). Each panel shows individual replicates (continuous lines), average values (dotted lines) and the CI_95_ of the simulated time course (shaded areas around dotted lines; these areas contain 95% of model outputs from MCMC iterations). It is apparent from this figure that when the *k* values were used to simulate the rate of Megalin arrival to SE^Rab4^, RE^TfR^ and RE^Rab11^, the simulated time courses fit smoothly with the experimental average time courses. Hence, we used them to simulate the fluxes of Megalin through all compartments of the model (Fig. 3f). As expected, the simulation showed that there is a large initial flux from PM to SE^Rab4^, which over time is nearly matched by the flux from SE^Rab4^ to RE^TfR^; the difference between these two fluxes is the small short-circuit flux from SE^Rab4^ back to the PM (Fig. 3f, left). Fluxes into and out of RE^TfR^ begin after a delay related to loading of SE^Rab4^; again the steady-state difference between the flux from SE^Rab4^ and the flux to RE^Rab11^ is referable to a small flux to the PM (Fig. 3f, middle). Fluxes into and out of RE^Rab11^ occur after the filling of RE^TfR^, and these are identical at steady-state (Fig. 3f, right). While the flux of Megalin throughout the system is uniform, its residence time in each compartment varies proportionally to the compartment size. Hence, as the relative endosome sizes are SE^Rab4^>RE^TfR^>RE^Rab11^, Megalin residence times are 622 s (CI_95_: 616–627 s), 353 s (CI_95_: 348–358 s) and 132 s (CI_95_: 129–134 s). The mathematical model confirms that internalized Megalin transits sequentially through SE^Rab4^, RE^TfR^ and RE^Rab11^ and reveals that the largest recycling fraction of Megalin to the PM originates from RE^Rab11^, with much smaller recycling fractions from SE^Rab4^ and RE^TfR^. These results demonstrate that (i) Megalin is a fast-recycling receptor; (ii) Like TfR and LDLR, internalized Megalin reaches SE^Rab4^ and RE^TfR^ and (iii) unlike TfR and LDLR, Megalin traffics through an additional endosomal compartment, RE^Rab11^, before return to the PM. Confocal live-imaging colocalization and mathematical modelling utilized in these experiments, combined, provide a precise measurement of the kinetics of Megalin arrival to each endosomal compartment. This approach constitutes an excellent tool to accurately and quantitatively measure the endosomal itinerary of Megalin or any endocytic receptor.

### Endosomal itinerary of Megalin in polarized MDCK cells

**Figure 4:**
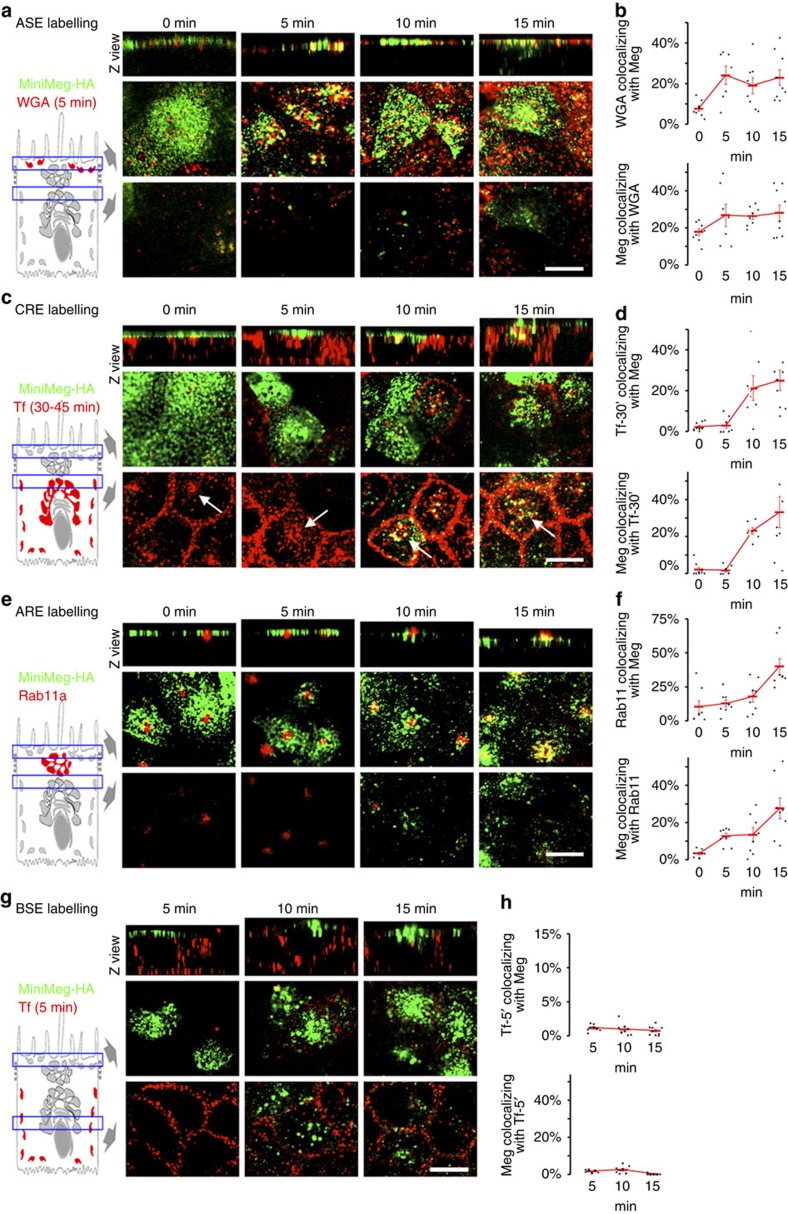
Megalin endosomal itinerary in polarized MDCK cells. mMeg-MDCK cells were polarized on Transwell filters and 488-MαHA pre-bound to the apical PM was allowed to internalize for the indicated times (apical internalization assay) (**a**) mMeg-MDCK cells subjected to the 488-MαHA apical internalization assay were labelled for ASE with 5 min apical incubation of 594-WGA followed by stripping of the 594-WGA bound to the apical PM with *N*-acetyl-D-glucosamine (10  min at 4 °C, three times). Each panel displays Z view (top) and confocal sections at the level of the apical PM (middle) and supranuclear region (bottom). (**b**) Quantification of the percentage of the ASE marker pixels co-localizing with the 488-MαHA pixels (top) and of the percentage of the 488-MαHA pixels colocalizing with the ASE marker pixels (bottom). Circles represent individual cells, red lines represent average and s.e.. (**c**) mMeg-MDCK cells subjected to the 488-MαHA apical internalization assay were labelled for CRE with basolateral incubation of 594-Tf applied for 30 min. CRE appear as a subpopulation of 594-Tf-positive endosomes localized in the supranuclear region (arrows). (**d**) Quantification of the percentage of the CRE marker pixels co-localizing with the 488-MαHA pixels (top) and of the percentage of the 488-MαHA pixels co-localizing with the CRE marker pixels (bottom). (**e**) mMeg-MDCK cells subjected to the 488-MαHA apical internalization assay were labelled for ARE with anti-Rab11 antibodies. (**f**) Quantification of the percentage of the ARE marker pixels colocalizing with the 488-MαHA pixels (top) and of the percentage of the 488-MαHA pixels co-localizing with the ARE marker pixels (bottom). (**g**) mMeg-MDCK cells subjected to the 488-MαHA apical internalization assay were labelled for BSE with 5 min basolateral incubation of 594-Tf. (**h**) Quantification of the percentage of the BSE marker pixels co-localizing with the 488-MαHA pixels (top) and of the percentage of the 488-MαHA pixels colocalizing with the BSE marker pixels (bottom). Scale bar, 10 μm.

Next, we studied the endosomal itinerary of Megalin in fully polarized MDCK cells (Fig. 4 and Supplementary Fig. 5). To this end, we used mMeg-MDCK cells confluent for 4 days on Transwell filters. We labelled mMeg-Ha on the apical surface at 4 ^o^C with a mouse anti-HA antibody tagged with CF-488 (488-MαHA), incubated for various times at 37 ^o^C to allow its internalization, fixed and processed the samples for dual colour analysis by spinning-disc confocal microscopy. Arrival of Megalin to specific endosomal compartments (for example, ASE, CRE, ARE and BSE, labelled with protocols that do not require overexpressing markers of these compartments^29^,^30^) was measured by quantitative co-localization (for detailed protocols see figure legends and Methods).

As ASE are the sorting endosomes associated with the apical PM (Fig. 1c), they are likely to be the first compartment visited by apically internalized Megalin. To test this hypothesis, we performed the 488-MαHA apical internalization assay in monolayers exposed to apical Alexa-594-tagged wheat germ agglutinin (594-WGA) for the last 5 min immediately before fixation. Since 595-WGA bound to the PM is stripped with *N*-acetyl-D-glucosamine, the remaining 594-WGA signal labels ASE^28^,^30^. Strikingly, whereas at *t*=0 min, 488-MαHA-decorated mMeg-HA distributed homogenously over the apical PM, at *t*=5 min, it decorated 594-WGA-positive subapical puncta (Fig. 4a,b; see split channels in Supplementary Fig. 5). This experiment shows that Megalin's first endocytic station in polarized MDCK cells is ASE.

It is currently unknown whether the endosomal itinerary of Megalin intersects the basolateral recycling pathway at CRE. Therefore, we performed the 488-MαHA apical internalization assay in cells incubated basolaterally with CF-594-tagged transferrin (594-Tf)^30^. The CRE compartment is easily identified as a cluster of perinuclear 594-Tf-positive endosomes (Fig. 4c, arrows). At *t*=0, 488-MαHA localized to the apical PM. At *t*=5 min it labelled subapical puncta (ASE) but did not co-localize with 594-Tf. Strikingly, after t=10 min, 488-MαHA progressively accumulated in the perinuclear region, where it robustly co-localized with 594-Tf (Fig. 4c,d). These experiments clearly demonstrate that the apical recycling pathway of Megalin intersects the basolateral recycling pathway of TfR at CRE.

Since the experiments in subconfluent MDCK cells indicated that Megalin visits a Rab11-positive recycling compartment after leaving RE^TfR^, we investigated whether Megalin traffics through Rab11-positive ARE after reaching CRE in polarized MDCK cells. To this end, mMeg-MDCK cells subjected to the 488-MαHA apical internalization assay were fixed and processed for double immunofluorescence with Rab11 antibodies. Colocalization of 488-MαHA with Rab11 was negligible at t=0 min, partially increased at 5 and 10 min and was maximal after 15 min (Fig. 4e,f), indicating that mMeg-HA reached ARE after leaving CRE.

Consistently with the fact that BSE is an endosomal compartment restricted to the basolateral recycling pathway, apically internalized mMeg-HA did not co-localize with BSE (identified through basolateral incubation with 594-Tf for 5 min) at any time point (Fig. 4g,h). These results demonstrate that Megalin sequentially traverses ASE, CRE and ARE, in very good agreement with the results of Fig. 3 in subconfluent MDCK cells.

### Polarized apical recycling of Megalin requires microtubules

**Figure 5:**
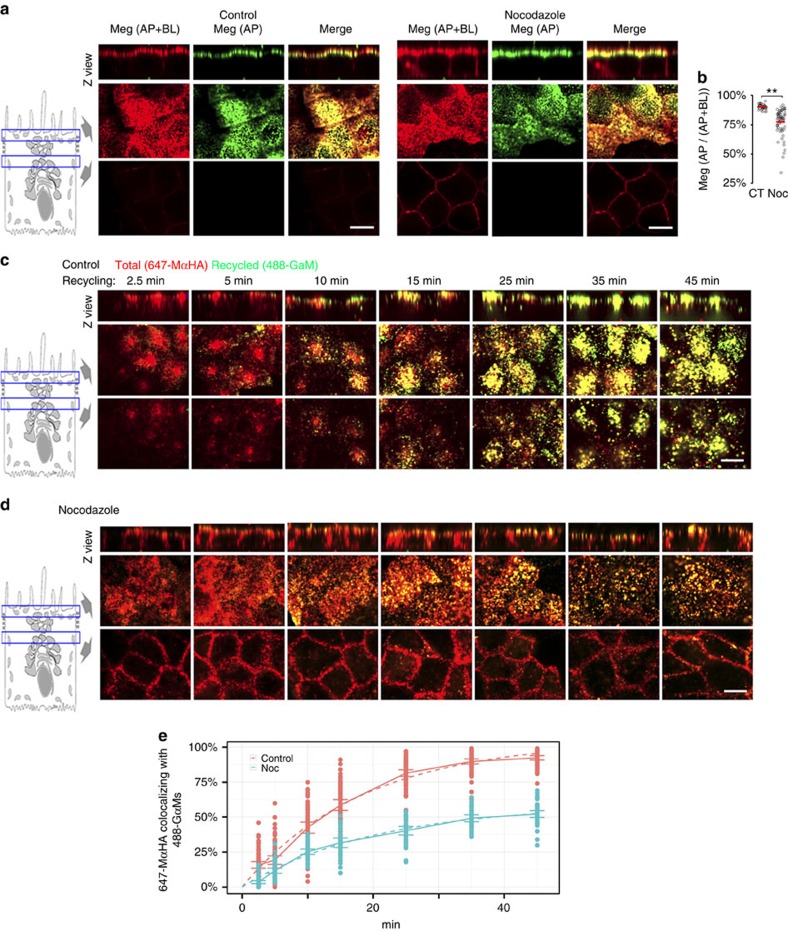
Microtubules mediate Megalin apical localization and recycling in polarized MDCK cells. (**a**) Confocal images of control and nocodazole-treated (2 h) mMeg-MDCK cells polarized on Transwell filters and stained with 647-MαHA for the basolateral mMeg-HA and with both 647-MαHA and 488-GαM for the apical mMeg-HA. Each panel displays Z view (top) and confocal sections at the level of the apical PM (middle) and supranuclear region (bottom). (**b**) Co-localization quantification of the percentage of the apical mMeg-HA pixels co-localizing with the surface mMeg-HA pixels, which informs Megalin Apical/Surface ratio. Circles represent individual cells, red lines represent average and s.e., **P<0.001, two-tailed Student's *t*-test. (**c**,**d**) Confocal images of control (**c**) and nocodazole-treated (**d**) mMeg-MDCK cells polarized on glass-bottom chambers, allowed to internalize 647-MαHA antibody for 90 min, washed and subsequently allowed to recycle for the indicated times in the presence of 488-GαM. (**e**) Co-localization quantification and fitted curve of the percentage of the 647-MαHA pixels co-localizing with the 488-GαM pixels, which informs the percentage of total mMeg-HA that was recycled to the apical PM at the indicated time points. Circles represent individual cells, the continuous lines represent average and CI_95_ and the dashed lines represent the fitted curves. Scale bar, 10 μm.

Microtubules mediate protein delivery to the apical PM in the biosynthetic^31^,^41^,^42^ and transcytotic^26^,^27^ routes; however their possible participation in the apical recycling pathway is poorly characterized as this epithelial pathway is itself not well defined. First, to study the role of microtubules in the apical localization of Megalin, we measured the apical/basolateral surface localization ratio of mMegHA in polarized mMeg-MDCK monolayers, control or treated with nocodazole for 2 h (Fig. 5a,b) (see figure legend and Methods for experimental details). Nocodazole promoted a small (15%) but statistically significant decrease in the percent of Megalin localized at the apical PM at steady state. Second, to study whether microtubules mediate the apical recycling of Megalin, we performed the mMegHa recycling assay described above (Fig. 2c) in control and nocodazole-treated polarized mMeg-MDCK cells (Fig. 5c,d). The results clearly showed that nocodazole treatment dramatically reduced mMeg-HA recycling, measured as the co-localization of previously internalized 647-MαHA with 488-GαM continuously added to the medium from *t*=0. Fitting of the co-localization data to a monoexponential function showed that the maximal co-localization plateau was reduced from 100%, CI_95_=100–101% in control cells to 59% (CI_95_=55–63%) in nocodazole-treated cells (*P*<0.001, Two-tailed Student's *t*-test) (Fig. 5e). Moreover, whereas at *t*=2.5 internalized Megalin strictly localized to apical and supranuclear endosomes in control cells, it was additionally detected in basolateral endosomes in nocodazole-treated cells (Fig. 5c,d and split channels in Supplementary Fig. 6), demonstrating that disruption of microtubules promotes basolateral missorting of mMeg-HA. As we previously reported that the kinesin KIF16B and the sorting lectin Galectin-4 mediate apical transcytosis of transferrin receptor^29^,^30^, we studied the role of these apical machinery components in the apical recycling of Megalin, using siRNA-mediated knock down. These experiments showed that KIF16B or Galectin-4 do not mediate apical recycling of Megalin (Supplementary Fig 7). In summary, these results demonstrate that the apical recycling of Megalin requires microtubules but the motors and sorting machinery involved are different from the machinery that mediates transcytosis of TfR. Furthermore, the mathematical analysis of Megalin recycling performed in these experiments allow us to conclude that the kinetics of Megalin recycling are similar in subconfluent and polarized mMeg-MDCK cells.

### Polarized apical recycling of Megalin requires Rab11

**Figure 6:**
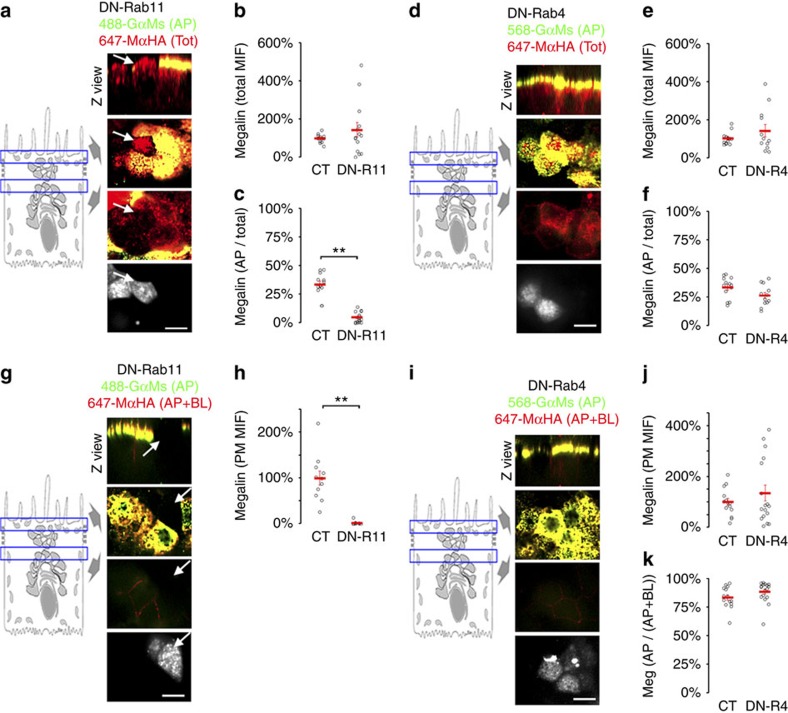
Rab11 mediates Megalin apical delivery in polarized MDCK cells. (**a**) Confocal images of mMeg-MDCK cells polarized on Transwell filters, transiently transfected with Ch-DN-Rab11 and stained with 647-MαHA for the basolateral and intracellular mMeg-HA and with both 647-MαHA and 488-GαM for the apical mMeg-HA. Each panel displays Z view (top), confocal sections at the level of the apical PM (middle-top), supranuclear region (middle-bottom) and a supranuclear confocal section displaying the signal of Ch-DN-Rab11 (bottom). Arrows denote Ch-DN-Rab11-transfected mMeg-MDCK cells. (**b**) Mean intensity fluorescence (MIF) of the total mMeg-HA. (**c**) Percentage of the apical mMeg-HA pixels co-localizing with the total mMeg-HA pixels (apical+basolateral+intracellular), which informs Megalin apical/total ratio. **P<0.001, two-tailed Student's *t*-test. Circles represent individual cells, red lines represent average and s.e. (**d**–**f**) Polarized mMeg-MDCK cells were transiently transfected with GFP-DN-Rab4 and subjected to equivalent experiments to those in **a**,**b** and **c**. (**g**) Confocal images of polarized mMeg-MDCK cells, transiently transfected with Ch-DN-Rab11 and stained with 647-MαHA for the basolateral mMeg-HA and with both 647-MαHA and 488-GαM for the apical mMeg-HA. (**h**) Mean intensity fluorescence (MIF) of the surface mMeg-HA pool (apical+basolateral). **P<0.001, two-tailed Student's *t*-test. (**i**,**j**) Polarized mMeg-MDCK cells were transiently transfected with GFP-DN-Rab4 and subjected to equivalent experiments to those in **g** and **h**). (**k**) Quantification of the percentage of the apical mMeg-HA pixels co-localizing with the surface (apical+basolateral) mMeg-HA pixels, which informs Megalin apical/surface ratio. Scale bar, 10 μm.

Rab11a has been identified as a very important component of the machinery that mediates biosynthetic delivery and transcytosis of PM proteins to the apical membrane^26^,^28^,^32^. Hence we studied the role of Rab11a in the steady-state localization and apical recycling of Megalin in polarized mMeg-MDCK cells. Transfection of a dominant negative Rab11 mutant (S25N) labelled with mCherry (Ch-DN-R11) decreased sevenfold the percent of total cell Megalin present at the apical surface (from 34±3 to 5±1, *P*<0.001, Two-tailed Student's *t*-test), (Fig. 6a,c and split channels in Supplementary Fig. 8a,c), with no decrease in the total amount of Megalin (Fig. 6b) or increase in the amount present at the basolateral surface (Fig. 6g,h). In contrast, similar experiments with the dominant negative S22N mutant of Rab4 labelled with GFP (GFP-DN-R4) showed no effect on mMeg-HA apical/total ratio and apical/basolateral ratio (Fig. 6d–f,i–k and split channels in Supplementary Fig 8). These experiments support a specific role of Rab11a in the apical trafficking of Megalin.

**Figure 7:**
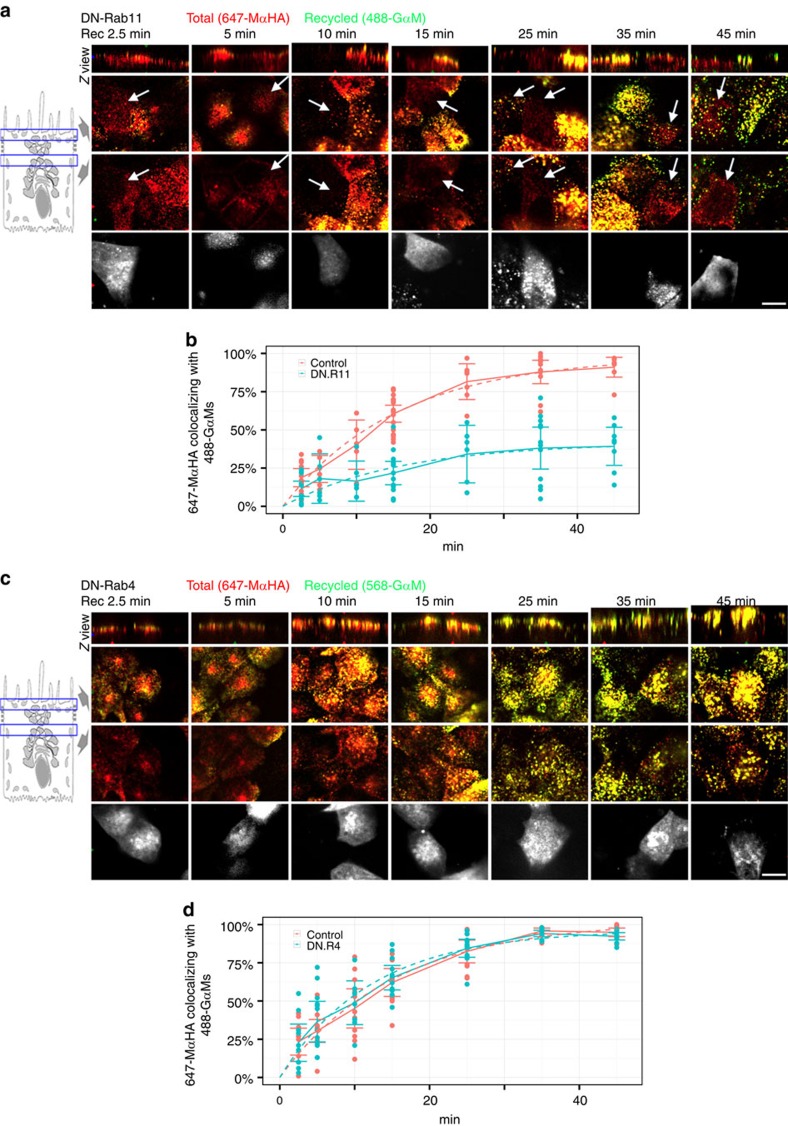
Rab11 mediates Megalin apical recycling in polarized MDCK cells. (**a**) Confocal images of mMeg-MDCK cells polarized on glass-bottom chambers, transiently transfected with Ch-DN-Rab11, allowed to internalize 647-MαHA antibody for 90 min, washed and subsequently allowed to recycle for the indicated times in the presence of 488-GαM. Each panel displays Z view (top), confocal sections at the level of the apical PM (middle-top), supranuclear region (middle-bottom) and a supranuclear confocal section displaying the signal of Ch-DN-Rab11 (bottom). Arrows denote Ch-DN-Rab11-transfected mMeg-MDCK cells. (**b**) Co-localization quantification and fitted curves in Ch-DN-Rab11-transfected and untransfected mMeg-MDCK cells from the same sample, for the percentage of the 647-MαHA pixels co-localizing with the 488-GαM pixels, which informs the percentage of total mMeg-HA recycled to the PM at the indicated time points. Circles represent individual cells, the continuous lines represent average and CI_95_ and the dashed lines represent the fitted curves. (**c**,**d**) Polarized mMeg-MDCK cells were transiently transfected with GFP-DN-Rab4 and subjected to equivalent experiments to those in **a** and **b**. Scale bar, 10 μm.

Direct evidence for a specific role of Rab11a in the apical recycling pathway of Megalin, was obtained by performing the recycling assay described in Fig. 2c in polarized mMeg-MDCK cells transfected with Ch-DN-R11 (or with GFP-DN-R4 as a control). Ch-DN-R11 caused a sevenfold decrease in the internalized 647-MαHA signal at *t*=2.5 (from 100±14 to 15±4, *P*<0.001, two-tailed Student's *t*-test). The 647-MαHA signal in Ch-DN-R11-expresing cells was high enough to quantify apical recycling of mMegHA, which was significantly inhibited by Ch-DN-R11, compared with untransfected cells from the same sample (Fig. 7a arrows and split channels in Supplementary Fig. 9). Fitting of the data to a monoexponential function showed that recycling of Megalin to the apical PM was reduced from 98%, CI_95_=90–101% in control cells to 42%, CI_95_=32–71% in Ch-DN-Rab11-expressing mMeg-MDCK cells (*P*<0.001, two-tailed Student's *t*-test) (Fig. 7b) but was not affected by GFP-DN-R4 (Fig. 7c,d). These results demonstrate a specific role of Rab11a in regulating apical polarity and recycling of Megalin.

## Discussion

The ability of Megalin to carry out its absorptive functions in epithelia, including the efficient and complete protein retrieval from the glomerular ultrafiltrate, requires that empty Megalin be delivered to the apical PM with the same rate as ligand-bound Megalin is internalized. Either the biosynthetic or the recycling pathways could potentially carry out this task; however, only the recycling pathway appears capable to efficiently provide the high Megalin levels required at the apical PM without wasteful Megalin loss. Notably, neither the kinetics nor the endosomal itinerary of Megalin recycling in epithelial cells have been fully characterized, in contrast with other fast-recycling receptors (for example, TfR and LDLR)^17^,^43^. Indeed, three recent papers have reported vastly different levels of Megalin recycling, ranging from virtually no recycling after 25 min to 80% recycling after 20 min (refs 37, 44, 45). In addition, the current model of Megalin recycling in polarized epithelial cells posits that Megalin recycles through a single endosomal compartment^4^. Therefore, we set out to characterize Megalin trafficking across all the major endosomal compartments of epithelial cells and obtain quantitative kinetic analysis of Megalin internalization, recycling and degradation. The results highlight novel and important aspects of Megalin's life-cycle and the apical recycling pathway of epithelial cells.

First, our results demonstrate that Megalin is a fast-recycling receptor like TfR and LDLR. The kinetics of internalization and apical recycling of Megalin are strikingly similar to those of the fast-recycling basolateral receptor TfR^17^,^43^. Our results in polarized MDCK cells show that Megalin is rapidly internalized from the apical PM, in agreement with previous reports^44^,^46^ and is then sequentially trafficked through ASE, CRE and ARE within approximately 5, 10 and 15 min, respectively. The mathematical model in subconfluent MDCK cells shows that most Megalin recycles to the PM from RE^Rab11^ (equivalent to ARE) with very limited recycling from SE^Rab4^ and RE^TfR^ (equivalent to ASE and CRE). Farquhar and co-workers^37^ also reported a low level of Megalin recycling from SE in non-epithelial L2 cells, which they attributed to interaction of NPxY sorting signals in Megalin's cytoplasmic domain with the clathrin adaptor ARH, that prevent interaction with Rab35 (required for recycling from SE) and promote downstream interactions with dynein, that mediate Megalin trafficking to RE. The very low levels of SE>PM recycling observed for Megalin contrasts with the high levels reported for the same step for TfR in non-epithelial cells^17^,^47^ and MDCK cells^43^; it has been suggested that this early recycling step for TfR is mediated by specific interactions with sorting nexin 4 (SNX4)^40^ rather than a default pathway, as originally suggested^17^. Farquhar and coworkers^37^ also reported Megalin overall PM recycling times of over 40 min, much longer than the 9 min in our experiments. Likely this reflects differences in the Megalin recycling assays used by the two laboratories. Whereas our recycling assay, based on the continuous addition of antibodies against mMeg-HA at 37 °C, can detect the large fraction of Megalin that is recycled (9 min) and rapidly re-internalized (1 min), surface biotinylation-based recycling assays, as used by Farquhar and co-workers, miss the re-internalized fraction and therefore overestimate recycling times.

Second, we report here that Megalin is a long-lived receptor, with a half-life slightly shorter than TfR. The concept that Megalin is a long-lived, fast-recycling receptor is consistent with a very efficient role of this receptor in protein reabsorption by the kidney PT and suggests novel mechanistic interpretations for proteinuric syndromes. For example, just a partial inhibition in Megalin recycling may account for the large absorptive effects observed in patients with recycling machinery mutations, for example, Lowe syndrome (PI4,5P_2_ 5-phosphatase OCRL) and Dent's disease (CLC-5) (refs. 45, 48, 49, 50). In light of our results, it will be interesting to examine whether the Megalin mutations that cause Donnai–Barrow syndrome and Cubilin mutations that cause Imerslund–Gräsbeck syndrome^13^,^14^,^16^ might lead to proteinuria by inhibiting the expression, apical PM delivery, binding to filtered proteins, endocytosis or recycling of these receptors.

Third, a major conclusion of this report is that in epithelial cells, Megalin recycles to the PM mainly from a specialized Rab11-positive RE compartment (RE^Rab11^), rather than from the Rab11-negative RE utilized by TfR and LDLR (RE^TfR^)^28^,^30^. Interestingly, in polarized epithelial cells this compartment is the ARE, a compartment intimately linked to trafficking to the apical PM of transcytotic proteins such as polymeric Ig receptor^26^,^28^ and newly synthesized apical proteins, such as p75, rhodopsin and endolyn^31^,^41^,^42^ (see model in Fig. 1c). Rab11-positive ARE have also been shown to play a key role in the *de novo* generation of the apical domain during epithelial morphogenesis in cysts^51^. These experiments highlight the importance of studying Megalin recycling in epithelial cells, as non-epithelial cells fail to generate two different classes of RE and recycle TfR to the PM from Rab11-positive RE. The mechanisms that epithelial cells utilize to generate two different classes of recycling endosomes are not known.

Fourth, we outline here an apical recycling pathway characteristic of epithelial cells that, to our knowledge, was incompletely defined by previous work. Comprehensive studies in MDCK cells have properly defined a major basolateral recycling pathway for TfR and LDLR^43^,^52^,^53^ and a basal to apical transcytotic pathway for pIgR^26^,^28^,^32^. In contrast, only fragments of a potential apical recycling pathway have been reported. Early experiments showed that apically internalized concanavalin A reaches a Tf-rich perinuclear compartment in Caco-2 cells and that apically localized pIgR is internalized into early apical endosomes compatible with ASE^21^,^54^; however, as discussed above for Megalin, the fate of these apically internalized proteins (recycling, transcytosis or degradation?) has remained obscure for over two decades. Our kinetic analysis unequivocally shows that after leaving ASE, Megalin mixes with basolaterally internalized TfR at perinuclear CRE and is subsequently translocated to ARE, from where it recycles to the apical PM in a Rab11- and microtubule-dependent manner. Therefore, our model posits that the apical recycling pathway is a three-endosome compartment route (Fig. 1c), as opposed to a single- or two-endosome compartment route proposed earlier. Our finding that Megalin intersects the basolateral recycling pathway at CRE suggests the possibility that a fraction of Megalin might transcytose to the basolateral membrane. Therefore our results are in line with a still controversial apical to basolateral transcytotic pathway for the recovery of albumin from the glomerular ultrafiltrate^10^. The apical recycling pathway we describe here is likely employed by other apical recycling receptors with key roles in embryo development, for example, Amnionless, and epithelial polarity, for example, Crumbs. Crumbs is recycled intracellularly through an Avalanche–Kybra–Retromer-mediated recycling pathway that is important to regulate a critical apical concentration required for its apical–basolateral polarity role^55^. Although the TGN was implicated in this apical recycling pathway, more detailed analysis may uncover a role for CRE, as this endosomal compartment lies geographically very close to the TGN.

Fifth, our experiments suggest that Megalin must use apical sorting mechanisms to exit CRE for delivery to ARE^53^,^56^,^57^. Sorting from CRE to ARE is a key step in the apical transcytotic pathway of pIgR in several epithelia and of TfR in epithelial cells lacking the basolateral sorting adaptor AP-1B. Previous work has shown that this step is mediated by *N*-glycan apical signals for pIgR and TfR and by the sorting lectin Galectin-4, the plus-end kinesin KIF16B and Golgi-nucleated microtubules in the case of TfR^26^,^27^,^29^,^30^. Here we show that Megalin apical recycling depends on microtubules, however, it does not appear to require KIF16B and Galectin-4, suggesting that Megalin's apical sorting machinery is different from that which mediates TfR apical transcytosis. Consistent with its endosomal itinerary, Megalin recycling kinetics are highly dependent on Rab11, but do not seem to depend on Rab4. The sorting machinery responsible for Megalin apical sorting remains to be elucidated. Lipid rafts, important components of the apical sorting machinery^58^,^59^, do not seem to be involved, since the association of Megalin with glycosphingolipids is apparently not required for its apical sorting^36^. Interestingly, previous work has shown that in the biosynthetic route Megalin is sorted apically by sorting motifs in its cytoplasmic tail, including a tyrosine motif^38^, suggesting the interesting possibility that clathrin and clathrin adaptors (currently implicated in basolateral sorting) might also be involved in the apical delivery of Megalin.

## Methods

### Cell culture

Wild-type MDCK cells (ATCC, Manassas, VA) were cultured in Dulbecco's MEM supplemented with 5% fetal bovine serum (Invitrogen, Carlsbad, CA), changed every 2 days. MDCK cells stably expressing the mMeg-HA construct (mMeg-MDCK) were selected and maintained in 0.2 mg ml^−1^ G418 (Mediatech, Manassas, VA). Subconfluent MDCK cells were plated at 1 × 10^5^ cells  cm^−2^ on 35 mm glass-bottom chambers and used 24 h later. Polarized MDCK cells were plated at 3 × 10^5^ cells cm^−2^ on either 12 mm Transwell filters or glass-bottom chambers and used 4 days later.

### Plasmids

The mMeg-HA plasmid was generated in Dr Maria Paz Marzolo's laboratory, the Rab4-GFP plasmids (WT and dominant negative) were kindly provided by Dr Marci Scidmore (Cornell University, Ithaca, NY). The TfR-GFP and Rab11-Cherry (WT and dominant negative) were described in our previous papers^29^,^30^.

### AMAXA electroporation

To transiently transfect plasmids, 4 × 10^6^ MDCK cells in suspension cultures were treated with 5 μg of the corresponding plasmid and electroporated with Amaxa Nucleofector kit V (program T23). Electroporated cells were plated at 1 × 10^5^ cells cm^−2^ on 35 mm glass-bottom chambers and used 24 h later.

### Buffers

Phosphate buffer saline (PBS): 137 mM NaCl; 2.7 mM KCl; 10 mM Na_2_HPO_4_; MgSO_4_, 5.3 mM KCl, 0.44 mM KH_2_PO_4_, 4.17 mM NaHCO_3_, 137.9 mM NaCl, 0.338 mM Na_2_HPO_4_, 5.55 mM dextrose. Hank's balanced salt solution (HBSS)–HEPES: HBSS supplemented with 20 mM HEPES. Lysis buffer: 40 mM Tris (pH 7.6), 150 mM NaCl, 1.5% Triton X-100.

### Antibodies and other reagents

The following antibodies were used: rabbit-αRab11 (715300. Invitrogen), mouse-αTfR (10R-CD71aHU. Fitzgerald, Acton, MA), mouse-αHA (MMS-101P. Covance, Princeton, NJ), chicken-αGAPDH (GW22763. Sigma-Aldrich, Saint Louis, MO), Alexa-labelled secondary antibodies (Invitrogen), IRdye-labelled secondary antibodies (Li-Cor, Lincoln, NE). The fluorophores used for protein conjugation were: SeTau-647 (SeTa Biomedicalos, Urbana, IL), CF-488 and CF-594 (Biotium, Hayward, CA). Alexa-594-labelled WGA was from Thermo Fischer (Grand Island, NY). Nocodazole was from Sigma-Aldrich.

### Transferrin and anti-HA labelling

Anti-HA antibodies were labelled with the fluorophores SeTau-647 or CF-488 (anti-HA), whereas iron-loaded human holo-Tf (Sigma-Aldrich) was conjugated with the fluorophore CF-594 (Tf), using the following procedure. Proteins were diluted in PBS pH 7.9 at 1 mg ml^−1^. NHS-fluorophores were diluted in anhydrous DMSO at 10 nmol μl^−1^. The NHS-fluorophore was mixed with the protein and rotated (60 min at room temperature). A 15 × fluorophore/protein molar ratio was used, which yields a theoretical ratio of three fluorophore/protein molecule. Fluorescent proteins were purified three times with 50 kDa cutoff centrifugal filters (Milipore, Billerica, MA). 594-Tf had been previously validated as a ligand for TfR through fluorescence microscopy experiments showing its co-localization with anti-TfR and through competition experiments that showed inhibition of 594-Tf uptake by the presence of 200 × unlabelled Tf^30^. Anti-HA antibodies were validated using WT and mMeg-HA expressing MDCK cells.

### Western blot

Cells were incubated in lysis buffer (30 min at 4 °C, mild shake) and centrifuged (30 min at 4 °C, max speed). Approximately 50 μg of protein samples were loaded in 4–12% gradient polyacrylamide pre-casted gels, ran (90 min, 100 mV) and transferred to nitrocellulose membrane using iBlot transfer stacks (Invitrogen). Representative uncropped blots are shown in Supplementary Fig. 2e.

### Surface biotinylation

mMeg-MDCK cells polarized on 12 mm Transwell filters were rinsed twice with cold (4 °C) HBSS. NHS-biotin (0.5 mg ml^−1^) in HBSS was added to either the apical or basolateral Transwell chamber in separate filters and incubated for 20 min (4 °C). This step was performed twice. Cells were incubated with 50 mM ammonium chloride in HBSS (20 min at 4 °C) and subsequently rinsed once with HBSS (4 °C). Filters were removed from their cases, placed on 24-well plates and lysed in 200 μl lysis buffer (30 min at 4 °C, mild shake). Lysates were centrifuged (30 min at 4 °C, 13,000 r.p.m.), 10% were separated for total lysate determination and the remaining was incubated with 50 μl Streptavidin-immobilized agarose beads (overnight at 4 °C). Pull-down and total lysate samples were mixed with 3 × Laemmli sample buffer and 1:20 BME and heated (30 min at 37 °C). The surface/total ratio was calculated as follows: (apical surface+basolateral surface)/[(apical total+basolateral total)/2 × 10]. The apical/basolateral ratio was calculated as follows: apical surface/(apical surface+basolateral surface) and apical surface/(basolateral surface+basolateral surface).

### Degradation assay

mMeg-MDCK were plated on 24-well plates at 1 × 10^5^ cells cm^−2^. Cells were treated for the indicated time with cycloheximide (100 μg ml^−1^) with or without E64 (5 μg ml^−1^) and Leupeptin (500 μg ml^−1^) (Sigma-Aldrich). Then, cells were lysed and processed for western blot analysis. Quantifications were done in Image J, by measuring the mMeg-HA/GAPDH and TfR/GAPDH ratios and normalizing to time 0.

### Surface binding and internalization assay

mMeg-MDCK cells either subconfluent or polarized were rinsed twice with cold HBSS–HEPES (4 °C) and incubated with 2.5 μg ml^−1^ 647-MαHA in HBSS–HEPES (45 min at 4 °C). In polarized MDCK cells 488-MαHA was applied only to the apical Transwell, unbound 488-MαHA was removed with three rinses with HBSS–HEPES followed by one incubation with HBSS–HEPES (15 min at 4 °C). To trigger endocytosis, cells were incubated with pre-warmed HBSS–HEPES (37 °C) for the indicated times, subsequently rinsed with cold (4 °C) HBSS–HEPES and fixed with 4% paraformaldehyde in PBS (10 min at 4 °C). Cells were incubated with 50 mM ammonium chloride in PBS (15 min at room temperature) followed by incubation with 5 μg ml^−1^ 488-GαM in PBS supplemented with 1% bovine serum albumin (1% BSA–PBS) (45 min at room temperature) and three washes with 1% BSA–PBS (5 min at room temperature).

### Recycling assay

Subconfluent mMeg-MDCK cells were rinsed twice with pre-warmed HBSS–HEPES (37 °C) and incubated with 2.5 μg ml^−1^ 647-MαHA in HBSS–HEPES (45 min at 37 °C). Unbound 647-MαHA was removed with three rinses with HBSS–HEPES followed by one incubation with HBSS–HEPES (15 min at 4 °C). To trigger recycling, cells were incubated with pre-warmed HBSS–HEPES (37 °C) for the indicated times in the presence of 5 μg ml^−1^ 488-GαM. Cells were subsequently rinsed three times and incubated for 15 min with cold HBSS–HEPES (4 °C) and fixed with 4% paraformaldehyde in PBS (10 min at 4 °C). Cells were incubated with 50 mM ammonium chloride in PBS (15 min at room temperature).

### Endosome labelling

Polarized mMeg-MDCK cells were subjected to: (i) the apical internalization assay of 488-MαHA described elsewhere in this section and (ii) one of the following endosome-labelling protocols ((ASE, CRE, ARE and BSE)).

ASE: polarized MDCK cells were incubated apically with 5 μg ml^−1^ 594-WGA in HBSS–HEPES (5 min at 37 °C). This solution was applied during the last 5 min of 488-MαHA apical internalization, except for time point 0, in which 594-WGA was applied before to the apical surface binding of 488-MαHA. BSE: polarized MDCK cells were incubated basolaterally with 5 μg ml^−1^ 594-Tf in HBSS-HEPES (5 min at 37 °C). This solution was applied during the last 5 min of 488-MαHA apical internalization, except for time point 0, in which 594-Tf was applied before to the apical surface binding of 488-MαHA. CRE: polarized MDCK cells were incubated basolaterally with 5 μg ml^−1^ 594-Tf in HBSS–HEPES (30 min at 37 °C). Then, cells were cooled to 4 °C to carry out the apical surface binding of 488-MαHA described elsewhere in this section. During this period, basolateral 5 μg ml^−1^ 594-Tf in HBSS–HEPES was applied (4 °C ). Next, cells were warmed to 37 °C to allow apical internalization of 488-MαHA. During this period, basolateral 5 μg ml^−1^ 594-Tf in HBSS–HEPES was applied (37 °C ). ARE: polarized MDCK cells were subjected to the apical internalization assay of 488-MαHA described elsewhere in this section, fixed with 4% paraformaldehyde in PBS (10 min at 4 °C) and incubated with 50 mM ammonium chloride (15 min at room temperature). Cells were blocked with 1% BSA–PBS (30 min at room temperature), incubated with anti-Rab11 antibody in 1% BSA–PBS (60 min at room temperature) followed by three washes with 1% BSA–PBS (5 min at room temperature). Then, cells were incubated with Alexa-568 anti-rabbit antibody in 1% BSA–PBS (40 min at room temperature) followed by three washes with 1% BSA–PBS (5 min at room temperature).

### Microscopy

Images were collected with a Zeiss Axio Observer inverted microscope, Yokogawa Confocal Scanner Unit CSU-X1, Rolera EMCCD and AxioCam-503 CCD cameras and Zeiss planapochromat × 63/1.4 oil-immersion objective.

### Co-localization analysis

Co-localization was quantified with the Manders' coefficients, which is one of the most accepted methods to measure co-localization between different cellular markers A and B^60^. To determine the area of marker ‘A' (that is, 647-MαHA) occupied by marker ‘B' (that is, 488-GαM), we quantified the pixels of marker ‘A' co-localizing with marker ‘B' divided by the total pixels of marker ‘A'. This operation was applied for all the confocal sections of a confocal stack in each region of interest (that is, one cell). The inverse formula was utilized to calculate the area of marker ‘B' occupied by marker ‘A'.

### Quantifications and mathematical analysis

Microscopy image quantifications was performed with Zen (Zeiss, Oberkochen, Germany) software. Western blot quantifications were performed with Image J. To fit the time course of Megalin endocytosis, and degradation we used a monoexponential decay function *y*=*y*_0_+*Ae*^−*x*/*t*^, whereas to fit Megalin recycling time course we used the *y*=*y*_0_+*Ae*^*x*/*t*^ exponential function, where *y*_0_=offset, *A*=amplitude, *t*=decay constant and *e*=2.71828 (the limit of (1+1 *n*^−1^)^*n*^ as *n* approaches infinity). *t*_1/2_ is calculated as 69% of the decay constant (*t*). Data are expressed as CI_95_.

### Mathematical model of Megalin endosomal itinerary

To represent the endosomal pathway of Megalin in subconfluent MDCK cells (experiment in Fig. 3), the pools of Megalin within PM, SE^Rab4^, RE^TfR^ and RE^Rab11^ are denoted *X*_0_, *X*_1_, *X*_2_ and *X*_3_, respectively. For a given experiment, the measure of Megalin (pixel density) within each compartment is denoted *x*(*i*,*t*), *i*=0,3, in which *t* is experimental time. In this model, Megalin transition from a donor to an acceptor compartment is assumed to be proportional to its density within the donor compartment, so that one can write linear differential equations for mass transfer. Corresponding to the cartoon of Fig. 3c are the equations:


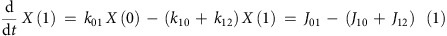



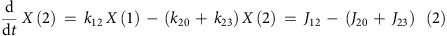



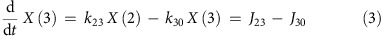


The constant coefficients, *k*_01_, *k*_12_, *k*_23_ and *k*_30_ denote transfer rates for sequential movement of Megalin through the compartments, while *k*_10_ and *k*_20_ correspond to short circuits from SE^Rab4^ and RE^TfR^ back to the PM. The terms on the right, *J*_*ij*_, denote the unidirectional fluxes between compartments. The system is completed with the requirement that over the time course of the experiments, the total Megalin pool of mass, *x*_*T*_, remains constant:


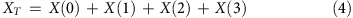


When equation (4) is used to eliminate *x*(0) from equation (1), the resulting equation is


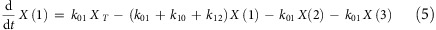


Equations (2), (3) and (5) are three linear equations with constant coefficients, which describe the model system. For the experiment of Fig. 3, it is assumed that all of the labelled Megalin starts within the PM, that is, for *t*=0, *x*(1)=*x*(2)=*x*(3)=0. Fitting this model to the data of Fig. 3 means solving for best values of the six model coefficients, *k*_*ij*_. For any set of choices of *k*_*ij*_, the model provides predictions for *x*(1,*t*), *x*(2,*t*) and *x*(3,*t*) for 0≤*t*≤*T*, where the experiment runs for time, *T*.

The goodness of fit was expressed as the error, *E*,


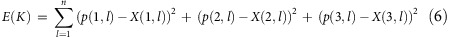


in which *p*(*i*,*l*) is the measured pixel density for compartment *i* at the *l* time point (displayed in Fig. 3), and *x*(*i*,*l*) is the model prediction for that value. This error, *E*(*K*), thus becomes a function of the six model coefficients, *k*_*ij*_ (denoted by the vector, *K*), and one seeks a set of coefficients that minimize this value. In the current work, this fit was determined in a three-step process. First a set of coefficients, *K*_0_, was determined empirically, which gave curves that resembled the experimental curves. In this search, a value of *x*_*T*_=2.0 was assumed, based on the absolute values for the pixel density curves. For the second step of the search, a grid was defined, which included 10 values for each coefficient that spanned two orders of magnitude above and two orders of magnitude below the empiric values of *K*_0_. Corresponding to 10 possible values for 6 parameters, this grid contained 10^6^ points, *K*, each corresponding to a distinct set of values for *k*_*ij*_. Using this grid, *E*(*K*) was evaluated 10^6^ times, and the vector, *K*_1_, was determined, which gave the smallest error. The third step of the search process was a steepest descent program, which recognized *E*(*K*) as a function of the six parameters, *k*_*ij*_, and sought a local minimum for *E*(*K*) at which all partial derivatives of *E* with respect to *k*_*ij*_ vanished. This steepest descent program was begun at the initial point, *K*_1_, identified in the search of step 2. The values identified by this third step appear in Fig. 3d. Of note, this steepest descent process only converged when the four sequential variables, *k*_01_, *k*_12_, *k*_23_ and *k*_30_ were used. This steepest descent procedure gave (meaningless) negative values for the short-circuit coefficients, *k*_10_ and *k*_20_, when these were included in the search, so for those two variables, their values from *K*_1_ were kept. The choice of the value for total Megalin, *x*_*T*_, reflected the scale of the pixel reporting. There was limited ability to find a good solution to the model system when *x*_*T*_ was varied away from 2.0. To ascertain the uncertainty in the optimized values for *k*_01_, *k*_12_, *k*_23_ and *k*_30_, a MCMC-based approach was used to generate an ensemble of credible sets of these parameters. Ten thousand iteration steps were generated using the modMCMC function within the R package FME^61^, and 95% confidence intervals for the model parameters were computed based on the resultant distributions. Results from a chain of 100,000 iterations (not shown) were nearly identical, indicating that the Markov chain was sufficiently long to produce converged distributions of the model parameters.

Figure 3e compares collected raw data with simulation results from an ensemble of credible models. Shaded area regions contain 95% of model outputs from MCMC iterations. The filled points show experimental averages at each time point, and solid lines show raw data for each experimental replicate. Although this figure shows that Megalin concentration within each compartment reaches a steady state, there are still substantial unidirectional Megalin fluxes through the system, and back to the PM. These fluxes correspond to the *J*_*ij*_ of equations (1)–(3), and are shown in Fig. 3f, in which the three panels again correspond to the three compartments. The flux units are pixels per second, and are determined by the experimental readings that define the Megalin concentrations in the three compartments. One way to factor out arbitrary pixel units is to consider the residence time, *τ*(*i*), for Megalin in compartment *i*, in which *J*(*i*) is the entering flux (that is, *J*_01_, *J*_12_ and *J*_23_ for SE^Rab4^, RE^TfR^ and RE^Rab11^, respectively).


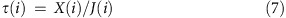


## Additional information

**How to cite this article:** Bay, A. E. P. *et al*. The fast-recycling receptor Megalin defines the apical recycling pathway of epithelial cells. *Nat. Commun.* 7:11550 doi: 10.1038/ncomms11550 (2016).

## Supplementary Material

Supplementary Information Supplementary Figures 1-9

Supplementary Movie 1 2D live-imaging movie of the subconfluent MDCK cell shown in Fig 3, transiently cotransfected with mMeg-HA and the SE marker Rab4-GFP and allowed to internalized surface-bound 647-MaHA during acquisition.

Supplementary Movie 2 2D live-imaging movie of the subconfluent MDCK cell shown in Fig 3, transiently cotransfected with mMeg-HA and the RE marker TfR-GFP and allowed to internalized surface-bound 647-MaHA during acquisition.

Supplementary Movie 3 2D live-imaging movie of the subconfluent MDCK cells shown in Fig 3, transiently cotransfected with mMeg-HA and the RE marker Rab11-Cherry and allowed to internalized surfacebound 647-MaHA during acquisition.

Supplementary Movie 4 3D live-imaging movie of the subconfluent MDCK cell shown in Supplementary Fig 4, transiently cotransfected with mMeg-HA and the SE marker Rab4-GFP and allowed to internalized surface-bound 647-MaHA during acquisition.

Supplementary Movie 5 3D live-imaging movie of the subconfluent MDCK cell shown in Supplementary Fig 4, transiently cotransfected with mMeg-HA and the RE marker TfR-GFP and allowed to internalized surface-bound 647-MaHA during acquisition.

Supplementary Movie 6 3D live-imaging movie of the subconfluent MDCK cell shown in Supplementary Fig 4, transiently cotransfected with mMeg-HA and the RE marker Rab11-Cherry and allowed to internalized surface-bound 647-MaHA during acquisition.
