# Supplementary material for: The fast-recycling receptor Megalin defines the apical recycling pathway of epithelial cells
Source: Nat Commun. 2016 May 16;7:11550. doi: 10.1038/ncomms11550 (PMC4873671; doi:10.1038/ncomms11550)
Supplement: Supplementary Information — Supplementary Figures 1-9 [file ncomms11550-s1.pdf]

Supplementary Information

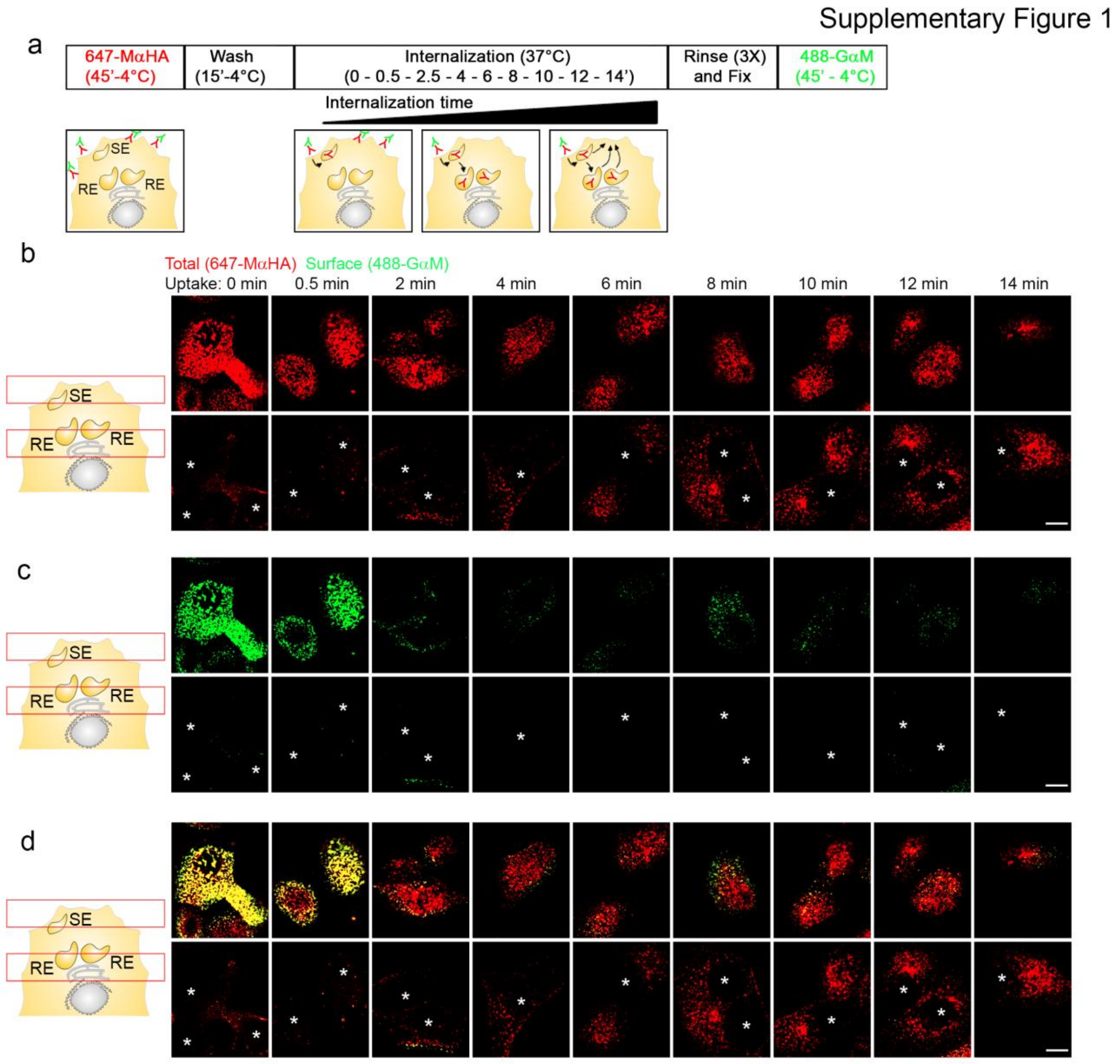

Supplementary Fig 1. Megalin endocytosis in subconfluent MDCK cells

(a) Schematic representation of mMeg-HA endocytic assay. Subconfluent mMeg-MDCK cells were labeled for the surface mMeg-HA with 647-MαHA (45'-4°C), unbound 647-MαHA was washed out (15'-4°C) and subsequently allowed to be endocytosed by shifting temperature to 37°C for the indicated times. Then, mMeg-MDCK cells were fixed and the PM pool of 647-MαHA was labeled with secondary 488-GαM antibody. **(b, c, d)** Confocal images of 647-MαHA **(b)**, 488-GαM **(c)** and Merge **(d)** signals of subconfluent mMeg-MDCK cells subjected to the protocol described in (a).The top row displays confocal sections in the upper part of the cells and the bottom row displays confocal section in the perinuclear region. Asterisks indicate the nuclei. Scale: 10 um.

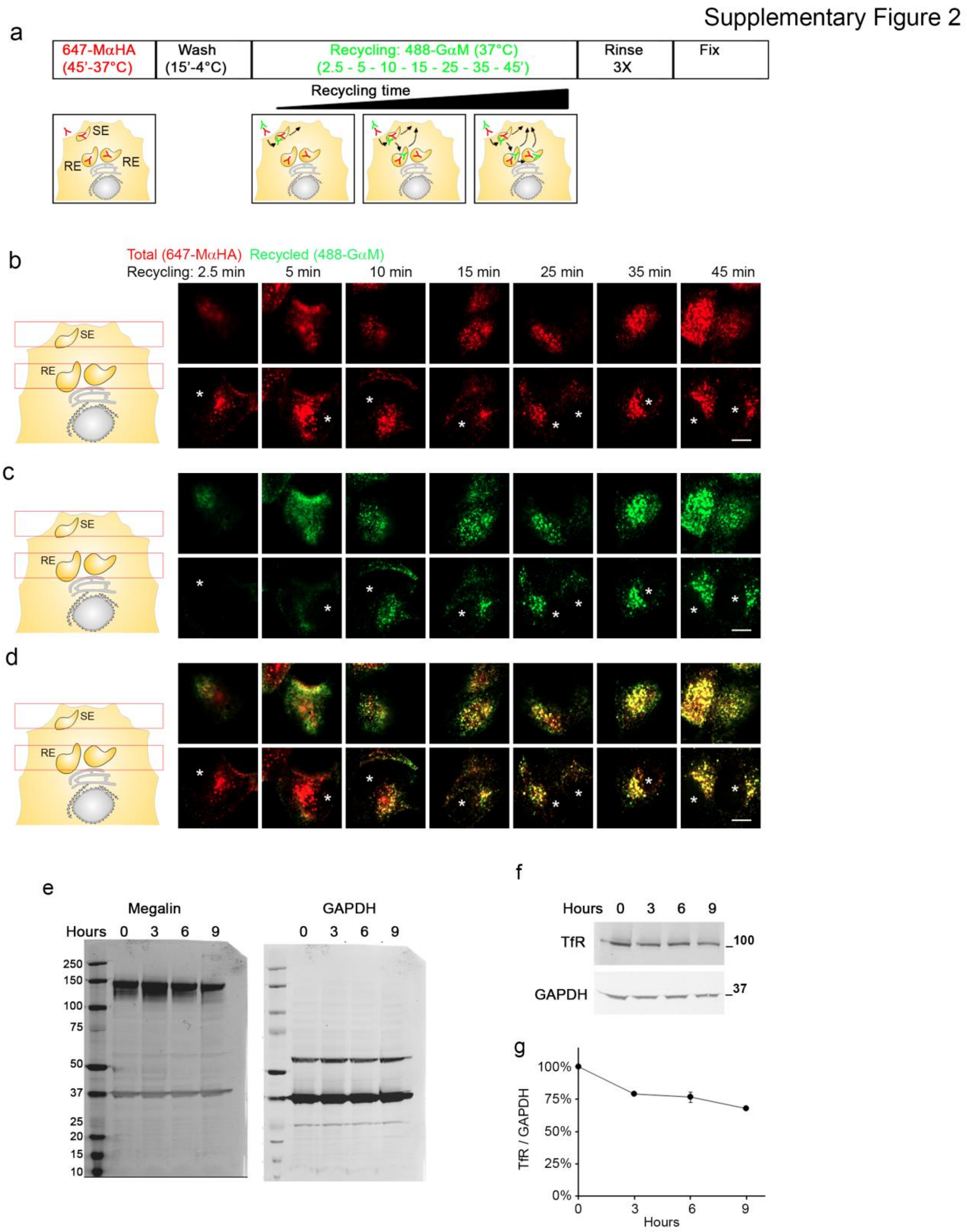

Supplementary Fig 2. Megalin recycling and TfR degradation in subconfluent MDCK cells

(a) Schematic representation of mMeg-HA recycling assay. Subconfluent mMeg-MDCK were incubated with 647-MαHA until antibody occupancy of all endosomal compartments stabilized (37°C-45 minutes), unbound 647-MαHA was washed out (15'-4°C) and internalized 647-MαHA was allowed to recycle by shifting temperature to 37°C for the indicated times. 488-GαM was applied during this period to label the fraction of 647-MαHA recycled to the cell surface. **(b, c, d)** Confocal images of 647-MαHA **(b)**, 488-GαM **(c)** and Merge **(d)** signals of subconfluent mMeg-MDCK cells subjected to the protocol described in (a). The top row displays confocal sections in the upper part of the cells and the bottom row displays confocal section in the perinuclear region. Asterisks indicate the nuclei. **(e)** Uncroped scans of the blots shown in Fig 2e. **(f)** Western blot analysis of TfR and GAPDH expression in subconfluent mMeg-MDCK cells treated with cycloheximide. **(g)** Quantification of the TfR/GAPDH ratio from two experiments as the one displayed in (a). Scale: 10 um.

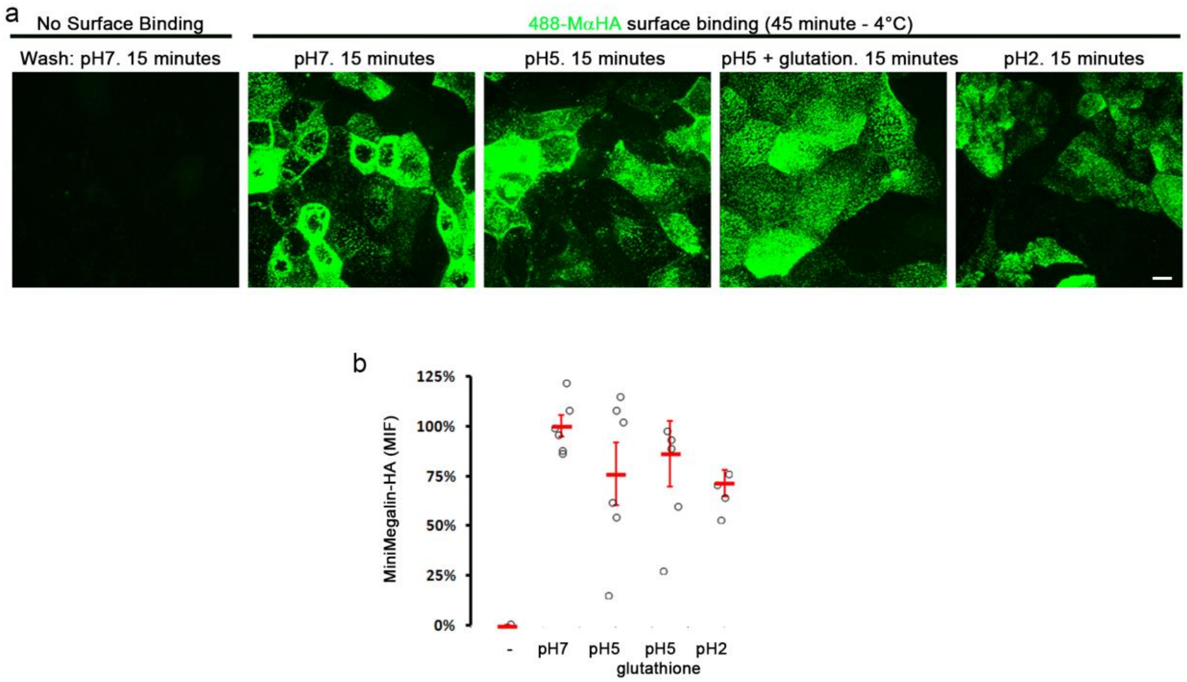

Supplementary Fig 3. 488-MαHA remains bound to the HA epitope under acidic environment **(a)** Subconfluent mMeg-MDCK cells were incubated with 488-MαHA (45’-4°C), rinsed once with Phosphate Balanced Solution, incubated for 15 minutes in the indicated washing conditions and rinsed again three times. **(b’)** Quantification of the mean intensity fluorescence of 488-MαHA after each washing condition. Circles represent individual cells, red lines represent average and standard error. Scale: 10 um.

Supplementary Figure 4

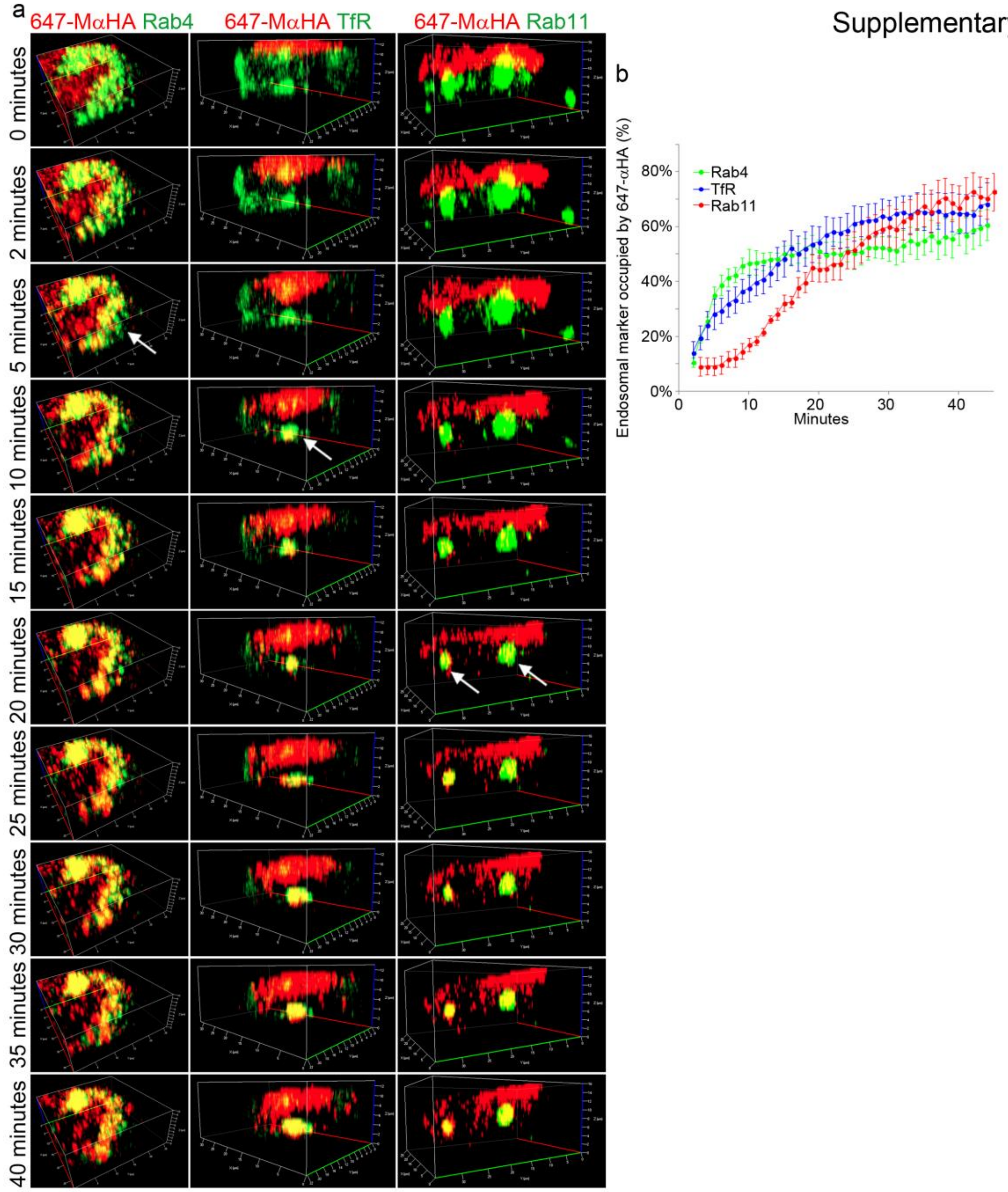

Supplementary Fig 4. Confocal 3D live-imaging shows Megalin endosomal itinerary in subconfluent MDCK cells

(a) Representative time-lapse 3D reconstitutions from confocal stacks of the cells shown in Fig 3. Subconfluent MDCK cells were transiently cotransfected with mMeg-HA and either the SE marker Rab4-GFP (left) or the RE markers TfR-GFP (middle) or Rab11-Cherry (right) and then allowed to internalize surface-bound 647-MαHA during 3D live-imaging acquisition. **(b)** Time course of the colocalization of

each endosomal marker with internalized 647-MαHA. Curves represent average and standard error values from six movies per condition.

Supplementary Figure 5

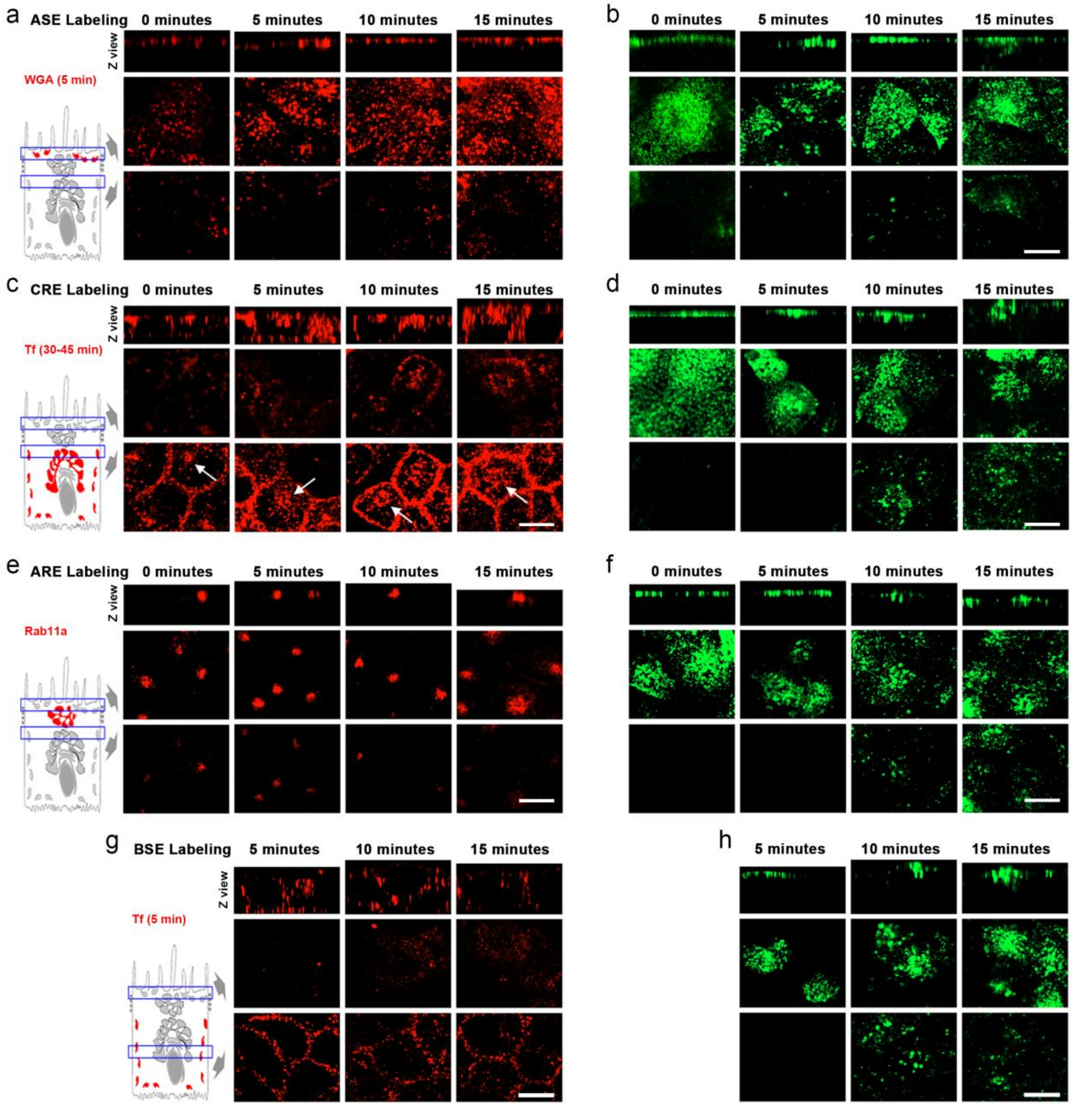

Supplementary Fig 5. Megalin endosomal itinerary in polarized MDCK cells

(a,c,e,g) Split confocal images from the images of Fig 4, showing polarized mMeg-MDCK cells labeled for: **(a)** ASE with 5 minutes apical incubation of 594-WGA, **(c)** CRE with 30 minutes basolateral incubation of 594-Tf (4°C) plus 0-15 minutes basolateral incubation of 594-Tf (37°C). CRE appear as

594-Tf-positive endosomes localized in the perinuclear region (arrows), **(e)** ARE with Rab11 immunostaining and **(g)** BSE with 5 minutes basolateral incubation of 594-Tf. **(b,d,f,h)** Split images from the images of Fig 4 showing the apical recycling time course of 488-MαHA-labeled-mMeg-HA at the indicated times for mMeg-MDCK cells labeled for **(b)** ASE, **(d)** CRE, **(f)** ARE and **(h)** BSE. Each panel displays Z view (top) and confocal sections at the level of the apical PM (middle) and perinuclear region (bottom). Scale: 10 um.

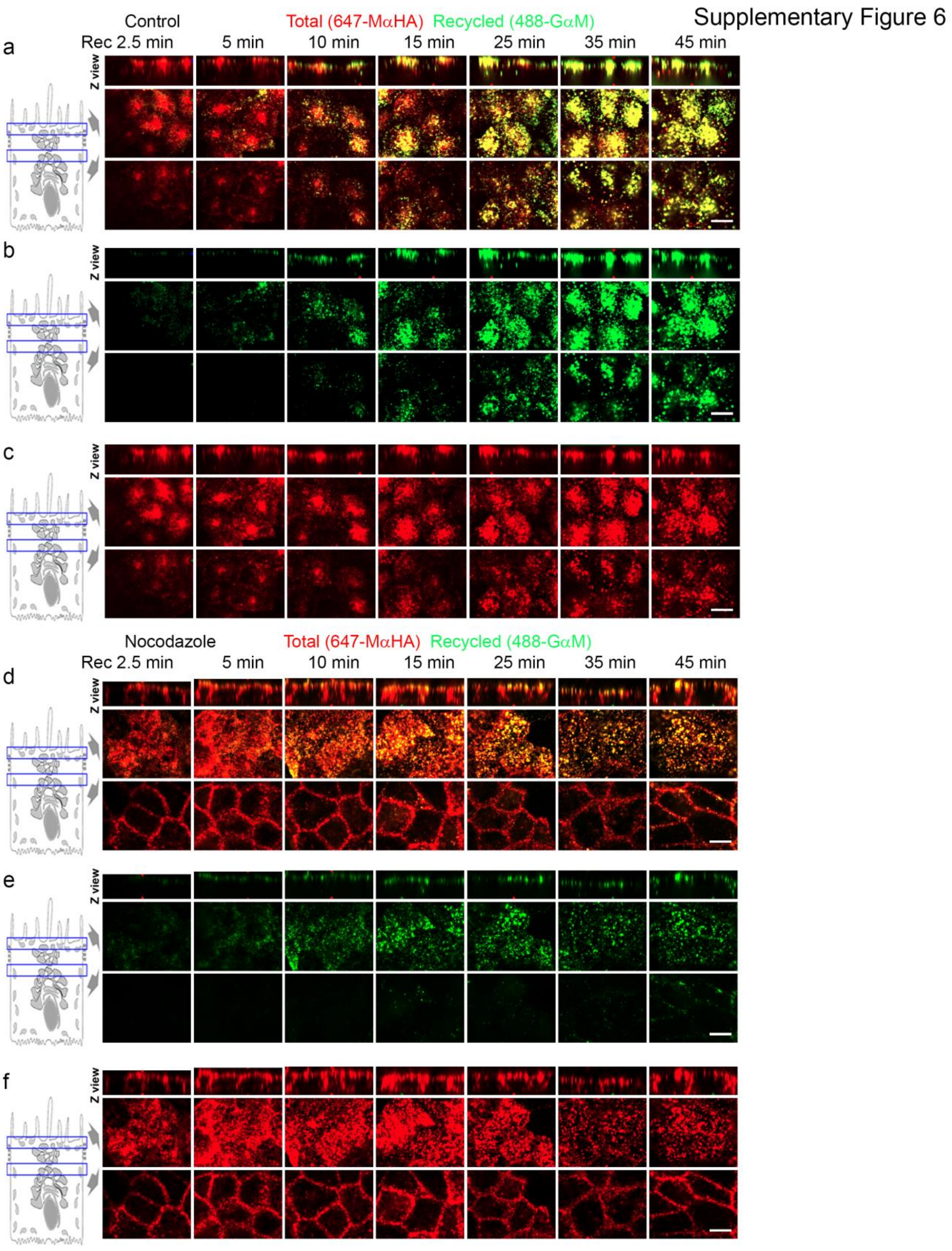

Supplementary Fig 6. Microtubules mediate Megalin apical recycling in polarized MDCK cells

Split channels from the confocal images of Figs 5b, c. Control **(a, b, c)** and nocodazole-treated **(d, e, f)** mMeg-MDCK cells polarized on glass-bottom chambers were allowed to internalize 647-MαHA antibody for 90 minutes and subsequently allowed to recycle for the indicated times in the presence of 488-GαM. Panels show the signals of 647-MαHA **(c, f)**, 488-GαM **(b, e)** and the Merge images **(a, d)**. Each panel displays Z view (top) and confocal sections at the level of the apical PM (middle) and supranuclear region (bottom). Scale: 10 um.

Supplementary Figure 7

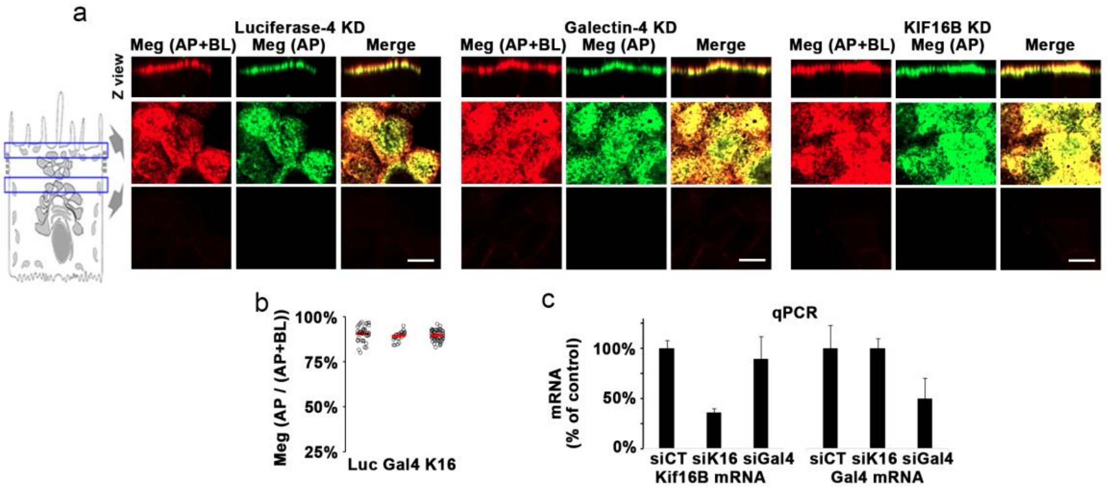

Supplementary Fig 7. Galectin-4 and KIF16B do not mediate Megalin apical sorting

(a) Confocal images of mMeg-MDCK cells polarized on Transwell® filters and knocked down for Luciferase (Left), Galectin-4 (Middle) or KIF16B (Right). The basolateral mMeg-HA was stained with 647-MαHA and the apical mMeg-HA with both 647-MαHA and 488-GαM. Each panel displays Z view (top) and confocal sections at the level of the apical PM (middle) and supranuclear region (bottom). **(b)** Quantification of the percentage of the apical mMeg-HA pixels colocalizing with the surface mMeg-HA pixels, which informs mMeg-HA Apical/Surface ratio. Circles represent individual cells, red lines represent average and standard error. **(c)** Quantitative PCR analysis for the expression levels of KIF16B and Galectin-4 in mMeg-MDCK cells polarized on Transwell® filters and knocked down for Luciferase, KIF16B or Galectin-4. Scale: 10 um.

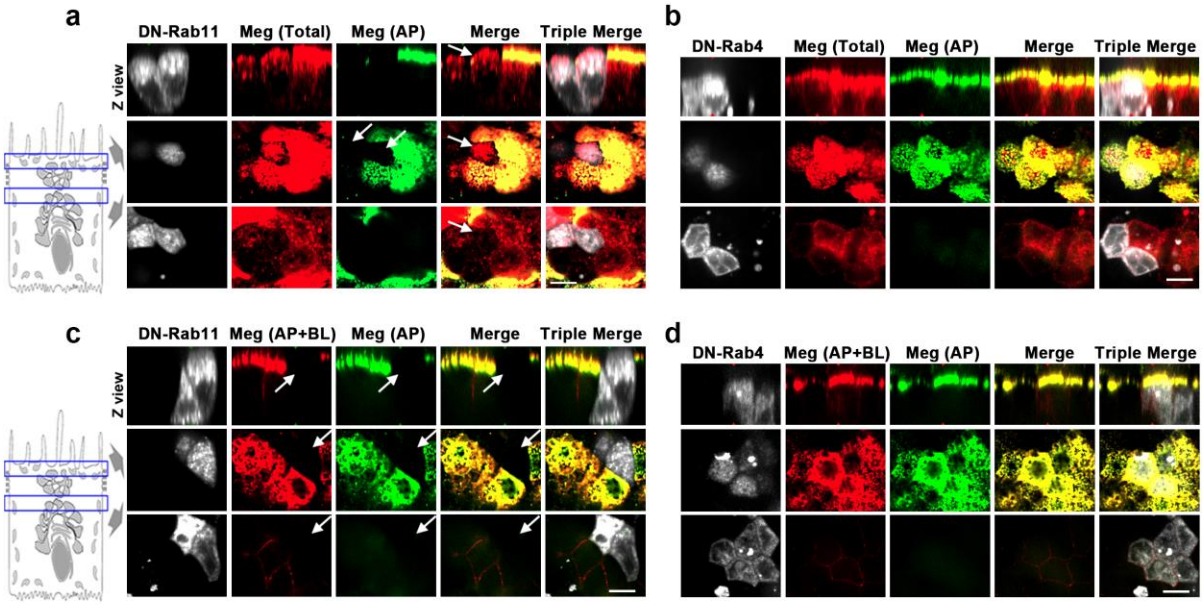

Supplementary Fig 8. Rab11 but not Rab4 mediates Megalin apical delivery in polarized MDCK cells

Split channels from the confocal images of Fig 6. **(a)** mMeg-MDCK cells polarized on Transwell® filters, transiently transfected with Ch-DN-Rab11 and stained with 647-MαHA for the basolateral and intracellular mMeg-HA and with both 647-MαHA and 488-GαM for the apical mMeg-HA. Each panel displays Z view (top), confocal sections at the level of the apical PM (middle) and supranuclear region (bottom). Arrows point to Ch-DN-Rab11-transfected mMeg-MDCK cells. **(b)** Polarized mMeg-MDCK cells were transiently transfected with GFP-DN-Rab4 and subjected to equivalent experiments to those in (a). **(c)** Confocal images of polarized mMeg-MDCK cells, transiently transfected with Ch-DN-Rab11 and stained with 647-MαHA for the basolateral mMeg-HA and with both 647-MαHA and 488-GαM for the apical mMeg-HA. **(d)** Polarized mMeg-MDCK cells were transiently transfected with GFP-DN-Rab4 and subjected to equivalent experiments to those in (c). Scale: 10 um.

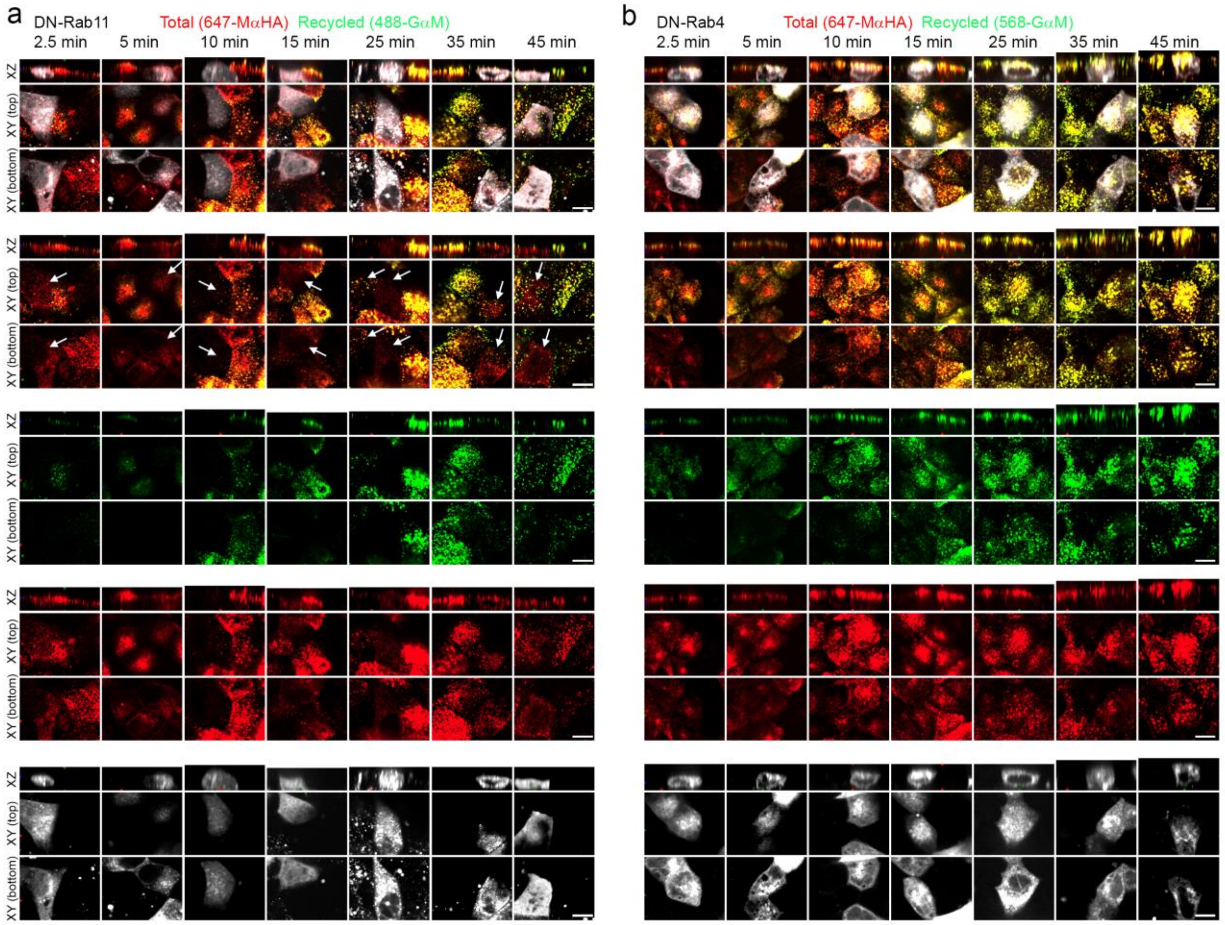

Supplementary Fig 9. Rab11 but not Rab4 mediate Megalin apical recycling in polarized MDCK cells

Split channels of the confocal images shown in Figs 7. **(a)** Confocal images of mMeg-MDCK cells polarized on glass-bottom chambers, transiently transfected with Ch-DN-Rab11, allowed to internalize 647-MαHA antibody for 90 minutes, washed and subsequently allowed to recycle for the indicated times in the presence of 488-GαM. Each panel displays Z view (top), confocal sections at the level of the apical PM (middle) and supranuclear region (bottom). Arrows denote Ch-DN-Rab11-transfected mMeg-MDCK cells. **(b)** Polarized mMeg-MDCK cells were transiently transfected with GFP-DN-Rab4 and subjected to equivalent experiments to those in (a). Scale: 10 um.
